# PRC2 loss drives MPNST metastasis and matrix remodeling

**DOI:** 10.1172/jci.insight.157502

**Published:** 2022-10-24

**Authors:** Qierra R. Brockman, Amanda Scherer, Gavin R. McGivney, Wade R. Gutierrez, Andrew P. Voigt, Alexandra L. Isaacson, Emily A. Laverty, Grace Roughton, Vickie Knepper-Adrian, Benjamin Darbro, Munir R. Tanas, Christopher S. Stipp, Rebecca D. Dodd

**Affiliations:** 1 Department of Internal Medicine,; 2 Molecular Medicine Training Program,; 3 Cancer Biology Training Program,; 4 Department of Molecular Physiology and Biophysics,; 5 Medical Scientist Training Program,; 6 Department of Ophthalmology and Visual Science,; 7 Department of Pathology,; 8 Department of Pediatrics, and; 9 Department of Biology, University of Iowa, Iowa City, Iowa, USA.

**Keywords:** Oncology, Cancer, Epigenetics, Mouse models

## Abstract

The histone methyltransferase PRC2 plays a complex role in cancer. Malignant peripheral nerve sheath tumors (MPNSTs) are aggressive sarcomas with frequent loss-of-function mutations in PRC2 that are associated with poor outcome. Here, we identify a critical role for PRC2 loss in driving MPNST metastasis. PRC2-dependent metastatic phenotypes included increased collagen-dependent invasion, upregulation of matrix-remodeling enzymes, and elevated lung metastasis in orthotopic mouse models. Furthermore, clinical sample analysis determined that PRC2 loss correlated with metastatic disease, increased fibrosis, and decreased survival in patients with MPNSTs. These results may have broad implications for PRC2 function across multiple cancers and provide a strong rationale for investigating potential therapies targeting ECM-remodeling enzymes and tumor fibrosis to improve outcomes in patients with MPNSTs.

## Graphical abstract

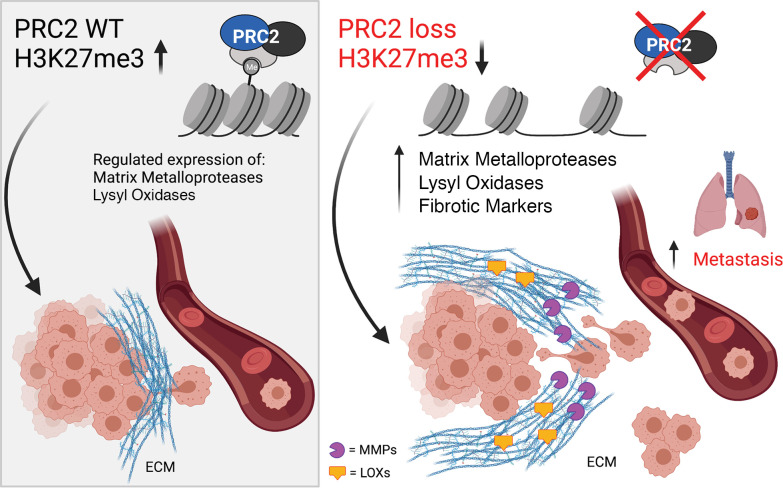

## Introduction

The histone methyltransferase PRC2 complex is a major epigenetic regulator of cancer progression that maintains chromatin silencing and cellular homeostasis through tri-methylation of histone H3 to lysine 27 (H3K27). Dysregulation of PRC2 activity is linked to poor prognosis, metastatic disease, and chemotherapy resistance across multiple cancers (1). PRC2 is composed of 3 core subunits: EED, SUZ12, and the catalytic subunit EZH2. All 3 subunits are required for histone methylation activity, although EED and SUZ12 play unique structural roles in PRC2 complex formation. Additionally, these subunits have autonomous functions in other pathways, such as EED’s role in PRC1 activity (2, 3) and SUZ12’s ability to regulate CpG binding in the absence of other PRC2 subunits (4). Intriguingly, the role of PRC2 in cancer is highly plastic and depends on tumor context (1). PRC2 functions as an oncogene in lung, ovarian, and prostate cancers; functions as a tumor suppressor in sarcoma, glioblastoma, and melanoma; and plays both roles in breast cancer and leukemia (1). Genetic disruptions in PRC2 can also drive metastasis through either activating (breast, prostate cancers) (5–8) or inactivating mutations (liver, oral squamous cell carcinoma) (9–11). Due to this functional complexity, the molecular and cellular events driving PRC2-regulated metastasis require careful examination within the context of each individual cancer.

Malignant peripheral nerve sheath tumors (MPNSTs) are aggressive, highly metastatic sarcomas that arise from the myelinating nerve sheath and are characterized by a dense, collagen-rich extracellular matrix (ECM). Up to 50% of MPNSTs metastasize to the lungs, and the 5-year survival rate of 20%–35% has not improved in the past 30 years (12–14). Genomic analyses of metastatic patient lesions have provided critical insight into several metastatic candidates, including TYK2 and TRIM23 (15, 16). However, the mechanisms driving MPNST metastasis remain poorly understood. MPNSTs have inactivating mutations in the tumor suppressor neurofibromin (*NF1*), a major regulator of Ras signaling. The majority of MPNSTs also have disruptions in the cyclin dependent kinase inhibitor 2A (*CDKN2A*) locus, though a small group has alternative mutations in the *p53* gene. Along with *NF1* and *CDKN2A*/*p53* disruption, approximately 60% of MPNSTs have loss-of-function mutations in *EED* or *SUZ12*, which are associated with worse overall survival (17–19). Importantly, limited patient data make it difficult to determine if EED and SUZ12 play functionally distinct or overlapping roles in MPNSTs. Despite several efforts examining PRC2 loss in MPNST biology (20, 21), the role of PRC2 in MPNST metastasis has not been explored to our knowledge, and it is not understood if EED and SUZ12 play unique or distinct roles in PRC2-dependent pathogenesis.

Metastatic dissemination requires disruption of multiple cellular processes to activate the metastatic cascade, which includes local invasion into the surrounding tissue, intravasation and survival in circulation, extravasation at the distant site, and proliferation at the new location. Modification of the ECM is one of the earliest steps in the metastatic process. ECM remodeling is a hallmark of tumor progression that involves activation of matrix-remodeling enzymes, including matrix metalloproteinases (MMPs) and lysyl oxidases (LOXs). While MMPs and LOXs are traditionally associated with ECM degradation, turnover, and stiffness, their main functions are in homeostatic regulation of the extracellular environment through activities such as proteolytic processing of signaling molecules (22–24). As such, there are complex crosstalk and compensatory mechanisms between matrix-remodeling enzymes that reflect their overlapping functions (25, 26). A role for PRC2 in matrix biology has been suggested by other groups, including increased fibrosis in an *Eed*-null lung cancer mouse model (27), EZH2-dependent remodeling of the embryonic endothelium, and regulation of protease activity through PRC2 binding to *Mmp* promoters (28, 29).

In this study, we use a combination of in vitro metastasis assays, 3D collagen studies, and orthotopic mouse models to examine mechanisms of metastasis in multiple paired, isogenic MPNST cells. This use of isogenic cell series allows for a direct comparison between *Suz12* and *Eed* loss in the same cells, in addition to examining these events in both *Cdkn2a*-null and *p53*-null genetic contexts. By combining these approaches with time-lapse microscopy and analysis of patient samples, we have determined a critical role for PRC2 loss in driving MPNST metastasis and ECM remodeling. Our data show that PRC2 deletion can increase lung metastasis in mouse models and identify a strong correlation in patient samples between PRC2 loss and increased metastatic disease. Furthermore, we demonstrate that PRC2 loss in MPNSTs induces expression of MMP and LOX family matrix-remodeling enzymes that drive metastatic phenotypes. This deeper understanding of PRC2-dependent metastatic events has a high potential to improve the clinical management of MPNSTs and has broad implications for PRC2 function across multiple cancers.

## Results

### Generation of isogenic PRC2-deleted MPNST cell line panels.

**Figure 1:**
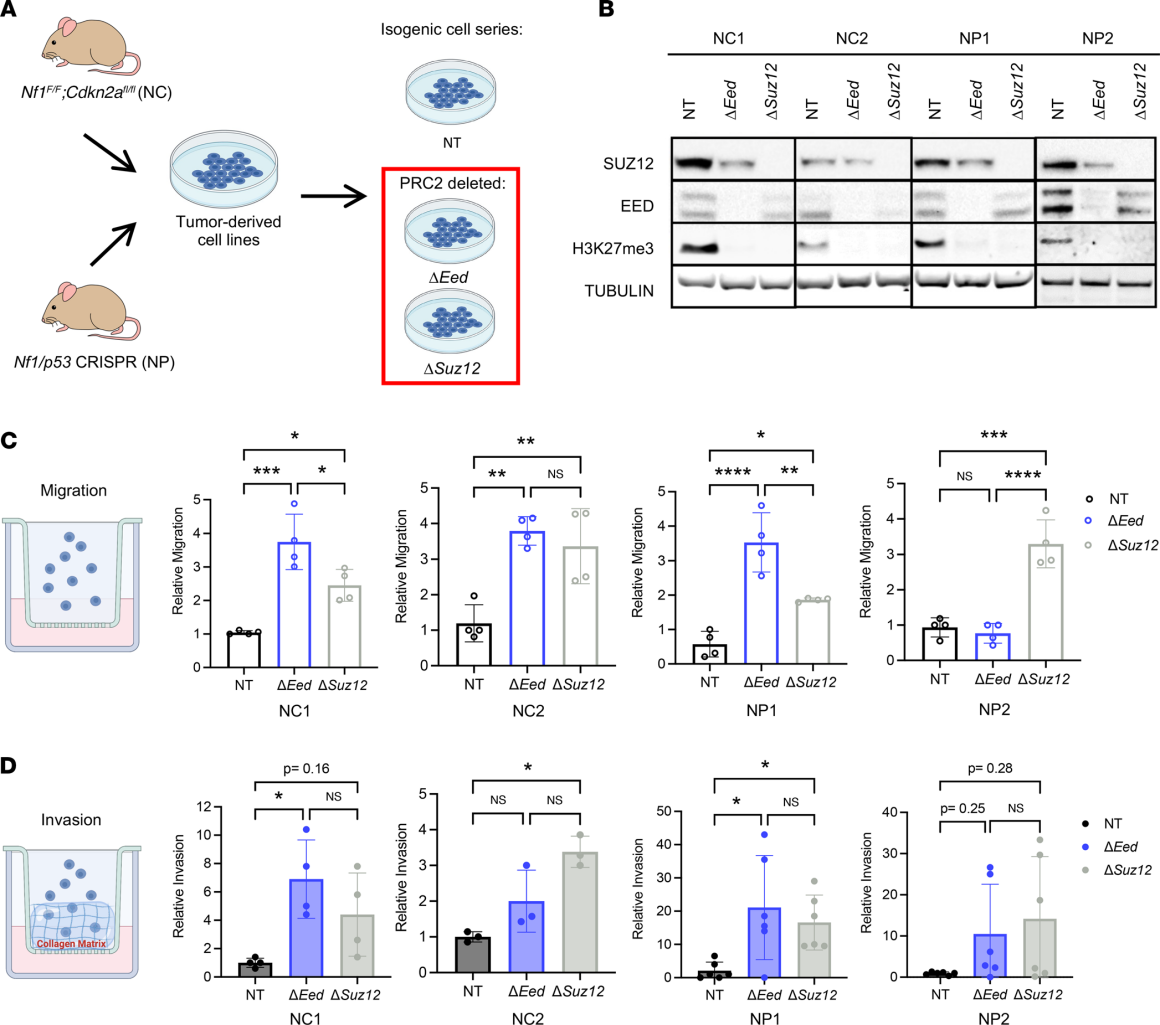
PRC2 deletion drives MPNST metastatic phenotypes in vitro. (**A**) The 4 parental cell lines were derived from primary mouse MPNST models generated from either NC tumors (*Nf1^fl/fl^ Cdkn2a^fl/fl^*) mice injected with adenovirus expressing Cre recombinaseinto the sciatic nerve) or NP tumors (wild-type mice injected with adenovirus containing Cas9 and guide RNAs for *Nf1* and *p53* into the sciatic nerve). Isogenic panels were made by CRISPR editing of cells in vitro with Cas9 and guide RNAs targeting *Eed*, *Suz12*, or a nontargeting control (NT). (**B**) Representative Western blots of isogenic cell series show loss of EED, SUZ12, and the downstream functional target H3K27me3 compared with NT (*n* = 3). (**C**) Transwell migration assays show increased migration of Δ*Eed* and Δ*Suz12* cells compared with NT by 1-way ANOVA with Tukey’s multiple comparisons (*n* = 4). (**D**) Transwell invasion assays show increased invasion through collagen of Δ*Eed* and Δ*Suz12* cells compared with NT by Kruskal-Wallis with multiple comparisons (*n* = 3–6). Data represent biological replicates with the mean ± SD; *P < 0.05, **P < 0.01, ***P < 0.001, ****P < 0.0001.

To study PRC2 loss in both *p53*- and *Cdkn2a*-null contexts, we used CRISPR/Cas9 to inactivate *Eed* or *Suz12* in murine MPNST cell lines derived from 2 complementary mouse models of spatially and temporally restricted MPNST (Figure 1A). These MPNST cell lines were generated from *Nf1/Cdkn2a*-deleted tumors (NC cells) or *Nf1/p53*-deleted tumors (NP cells). Both NC1 and NC2 cells originated from traditional Cre/*loxP* approaches that delivered adenovirus expressing Cre recombinase into the sciatic nerve of *Nf1^fl/fl^ Cdkn2a^fl/fl^* animals to generate *Nf1/Cdkn2a*-null MPNSTs (NC tumors) (30–32). To generate NP1 and NP2 cells, we applied our newly developed CRISPR/Cas9 somatic tumorigenesis technology to deliver adenovirus containing Cas9 and guide RNAs for *Nf1* and *p53* (NP tumors) (33, 34). By using these parallel genetic approaches, we can perform complementary studies that explore the unique combination of *p53* and *Cdkn2a* genetic mutations found in MPNST.

For each of these 4 parental lines, we generated a panel of isogenic cells that are deleted for *Eed* or *Suz12* or maintain wild-type PRC2 through delivery of a nontargeting guide RNA (nontargeting cells receiving “Tomato” control, which targets a fluorescent Tomato gene not expressed in the cells). Deletion of *Eed* or *Suz12* was verified by sequencing and Western blot for PRC2 subunits and tri-methylated H3K27 (H3K27me3), the downstream target of PRC2 activity (Figure 1B). Deletion of *Eed* resulted in loss of detectable H3K27me3 and EED protein, in addition to decreased levels of SUZ12 protein in all 4 isogenic groups. Similarly, *Suz12* deletion resulted in loss of H3K27me3 and SUZ12 protein, with reciprocal downregulation of EED subunits, which was more prominent in the NC cells. PRC2 loss had minimal impact on cell growth (Supplemental Figure 1; supplemental material available online with this article; https://doi.org/10.1172/jci.insight.157502DS1). These cells represent a tool kit of 4 paired isogenic lines, each with distinct PRC2 disruptions and different combinations of patient-relevant tumor suppressors.

### PRC2 deletion drives increased cell migration, invasion through collagen matrix, and cell clustering.

Loss of PRC2 is associated with poor outcome in patients with MPNSTs, and PRC2 disruption is associated with metastatic growth in colon, breast, and lung cancers (1). We hypothesized that loss of PRC2 would drive in vitro metastasis-associated phenotypes in MPNST cells. First, we tested the ability of *Eed* or *Suz12* deletion to affect migration across a Transwell membrane (Figure 1C). Serum-starved cells were plated above a 5% serum gradient, and cell number was counted after 4–6 hours. Loss of *Suz12* was sufficient to induce migration in all 4 isogenic series. Similarly, *Eed* loss resulted in increased migration of NC1, NC2, and NP1 cells compared with nontargeting controls. To determine how PRC2 influences invasion through a collagen matrix, we coated Transwells with collagen and allowed cells to invade for 4–6 hours (Figure 1D). Cells with wild-type PRC2 displayed low levels of basal invasion through the collagen matrix. Robust invasion was observed with Eed loss in NC1 and NP1 cells, with trending increases in NC2 and NP2 cells. In Δ*Suz12* cells, invasion through the collagen matrix was increased in 3 of the 4 cell panels.

**Figure 2:**
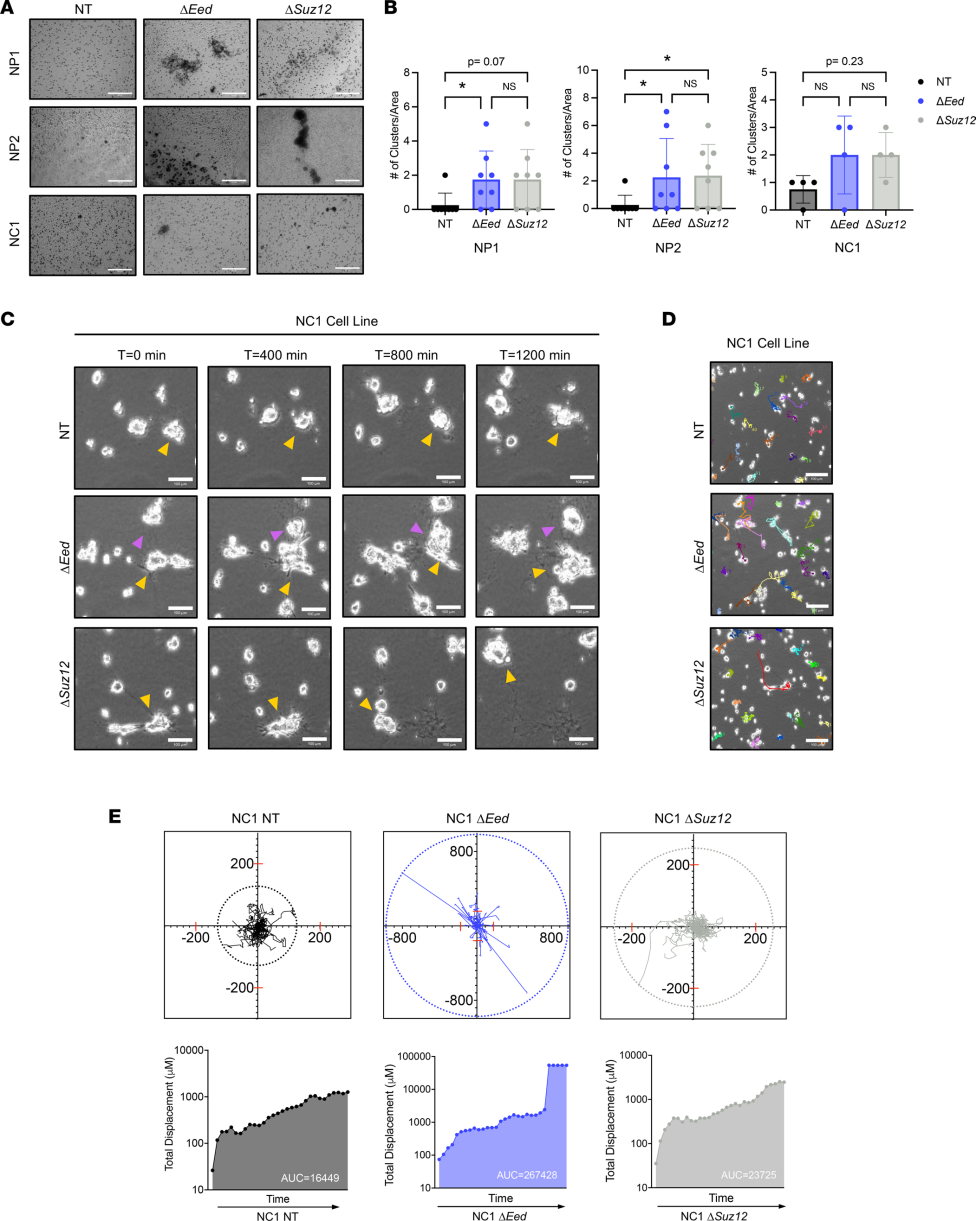
PRC2 deletion alters interaction with collagen matrix. (**A**) Representative images (original magnification, 20×) of NP1, NP2, and NC1 cell panels showing clustering phenotype observed after Transwell invasion (*n* = 4). (**B**) Quantification of cell clusters from Transwell invasion assays showing increased number of clusters with PRC2 loss. Total number of clusters was analyzed by Kruskal-Wallis with multiple comparisons. (Area = 0.546 mm^2^) (*n* = 4). (**C**) Phase-contrast movies of NC1 cells plated on collagen for 18 hours before capturing cell dynamics via 5-minute increment frames for 20 hours (1,200 minutes; original magnification, 10×). Yellow and pink arrows denote key cell motility features of the same cell across multiple frames. Little movement is seen in the NT cells, while the formation of cell clusters (Δ*Eed* frames) and rapid movement (Δ*Suz12* frames) are observed with PRC2 loss (*n* = 3). (**D**) Representative images of movies (original magnification, 10×) with cell motility tracks highlighted. (**E**) Cell motility tracks (*n* = 25) plotted on an *x*/*y* coordinate graph illustrate total displacement by color ring of NT (left), Δ*Eed* (middle), and Δ*Suz12* (right). Quantification of total displacement over time is calculated with total displacement (μM) using area under the curve analysis. Scale bars at 200 μm. Data represent individual clusters with the mean ± SD; *P < 0.05.

During close inspection of the collagen-coated Transwell membranes (the side representing productive invasion), we observed multicellular clusters composed of cells in direct contact with each other in the NC1, NP1, and NP2 series (Figure 2A and Supplemental Figure 2A). To further explore the formation of these PRC2-regulated clusters, we quantified the number of clusters of invading cells and determined these structures formed predominantly in PRC2-deleted cells (Figure 2B and Supplemental Figure 2, B and C). Furthermore, these features were only present in invasion studies where cells moved through collagen and were not observed during migration studies when a matrix was not present. Other groups have reported the formation of cell clusters during interactions with and movement through the matrix due to physical pressures or cellular signaling induced by cell-matrix contact (35, 36). Indeed, the formation of collective cellular clusters has been observed during metastatic dissemination of several epithelial cancers, including breast and lung, and this collective migration phenotype is associated with increased formation of circulating tumor cells in patient samples (37–40).

To further explore these phenotypes, we generated time-lapse movies of low-density MPNST cells plated on a collagen matrix (Figure 2, C and D, and Supplemental Videos 1 and 2). Over 24 hours of movement, we observed PRC2-deleted cells migrating toward each other, forming clusters, and deforming the surrounding matrix by possibly exerting tension on collagen fibers. In contrast, these features were absent in control cells with wild-type PRC2, which showed minimal movement and existed predominantly as single cells. PRC2-null cells were observed migrating long distances and merging into clusters, as illustrated by the yellow arrows highlighting the behavior of individual clusters across multiple frames (Figure 2C). To further examine the migration patterns of PRC2-deleted cells, coordinates from individual cell migration tracks were plotted for each genotype. We observed that both *Eed*- and *Suz12*-deleted cells had a greater net maximal displacement and total displacement from their origin (Figure 2E) compared with wild-type controls. Furthermore, quantification of these movements shows higher migration speeds and distance traveled in Eed-deleted cells compared with wild-type controls (Supplemental Figure 3). Taken together, these data demonstrate that loss of PRC2 drives multiple in vitro metastasis-associated phenotypes, including migration, invasion, speed, and cellular clustering in a matrix-dependent manner.

### PRC2 loss increases lung metastatic colonization in vivo.

**Figure 3:**
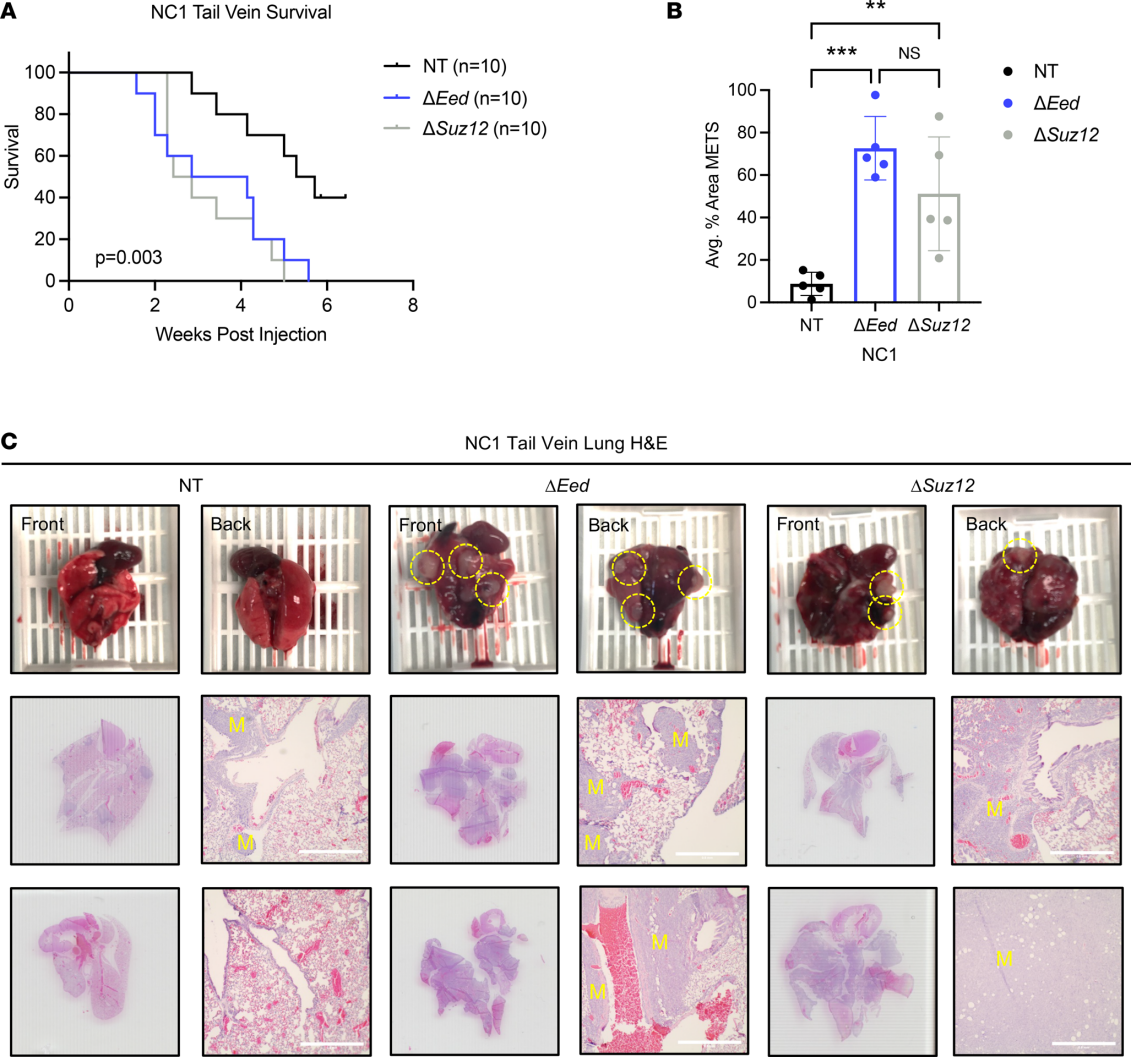
PRC2 loss increases metastatic colonization in vivo. (**A**) Decreased survival of NSG mice receiving tail vein injections of NC1 cells with loss of *Eed* (*n* = 10) or *Suz12* (*n* = 10) compared with NT (*n* = 10) cells with wild-type PRC2. (**B**) Increased metastatic lung area in mice receiving Δ*Eed* (*n* = 5) and Δ*Suz12* (*n* = 5) cells compared with NT (*n* = 5). Metastatic (MET) area quantification was performed on 5 lungs per group. Histological sections were taken at 0 μm, 100 μm, 200 μm, and 300 μm depths, and 4 images per section were taken at 4× objective. Data were analyzed using 1-way ANOVA with Tukey’s multiple comparisons. (**C**) Scans and 4× objective of 2 representative lungs from tail vein injections for each genotype. Metastases are indicated with a yellow “M.” Scale bars at 200 μm. Data represent biological replicates with the mean ± SD; **P < 0.01, ***P < 0.001.

Based on these in vitro data identifying PRC2-regulated MPNST metastatic phenotypes, we hypothesized that PRC2 deletion would increase metastatic potential in vivo. We injected the NC1 isogenic panel into the tail vein of NSG mice to determine the impact of PRC2 deletion on animal survival and the formation of lung metastases. Mice were euthanized when displaying signs of respiratory distress, including labored breathing, hunched posture, and ruffled fur. Lung metastasis was confirmed by gross examination and evaluation of H&E-stained lung sections. In tail vein–injected mice, deletion of either *Eed* or *Suz12* severely decreased metastasis-free survival (Figure 3A). Metastatic lung area was significantly increased in mice receiving PRC2-deleted cells, with an average of 70% and 50% of total lung area composed of metastatic nodules for Δ*Eed* and Δ*Suz12* tumors, respectively (Figure 3B). Upon gross examination, lungs from mice receiving PRC2-deleted cells revealed extensive, large, opaque metastases that commonly involved both lobes of the lung (Figure 3C). Histologically, metastases occurred throughout the lung, most noticeably penetrating deep into the lung interior and frequently observed in direct contact with surrounding blood vessels. While metastases were present in mice injected with control cells, these were significantly smaller and restricted to the outer surface of the lung. Combined with cell migration and invasion data above, these observations suggest that loss of either PRC2 subunit is sufficient to drive a robust and aggressive metastatic phenotype in MPNSTs. To determine if the elevated number of metastases was due to increased growth potential of PRC2-deficient cells at the metastatic site or was due to increased homing and/or lodging of PRC2-deficient cells in the lung itself, we performed a parallel study with fluorescently labeled cells at early time points. MPNST cells retained quantifiable CFSE signal up to 72 hours after labeling, with similar labeling across PRC2 genotypes (Supplemental Figure 4). Twenty-four hours after CFSE labeling, cells were injected into the tail vein of mice, and lungs were harvested 24 hours later (*n* = 5–6 mice per genotype). Flow cytometry determined total number of remaining MPNST cells in the lung at these early time points (Supplemental Figure 5). These data showed that PRC2-deleted cells were found in the lung at levels similar to PRC2 wild-type cells at this critical early time point, suggesting PRC2 does not play a role in early cell homing from the blood to metastatic sites, but instead increases growth of cells once they have reached the lung.

### ECM-remodeling enzymes are elevated in PRC2-deleted MPNST cells.

**Figure 4:**
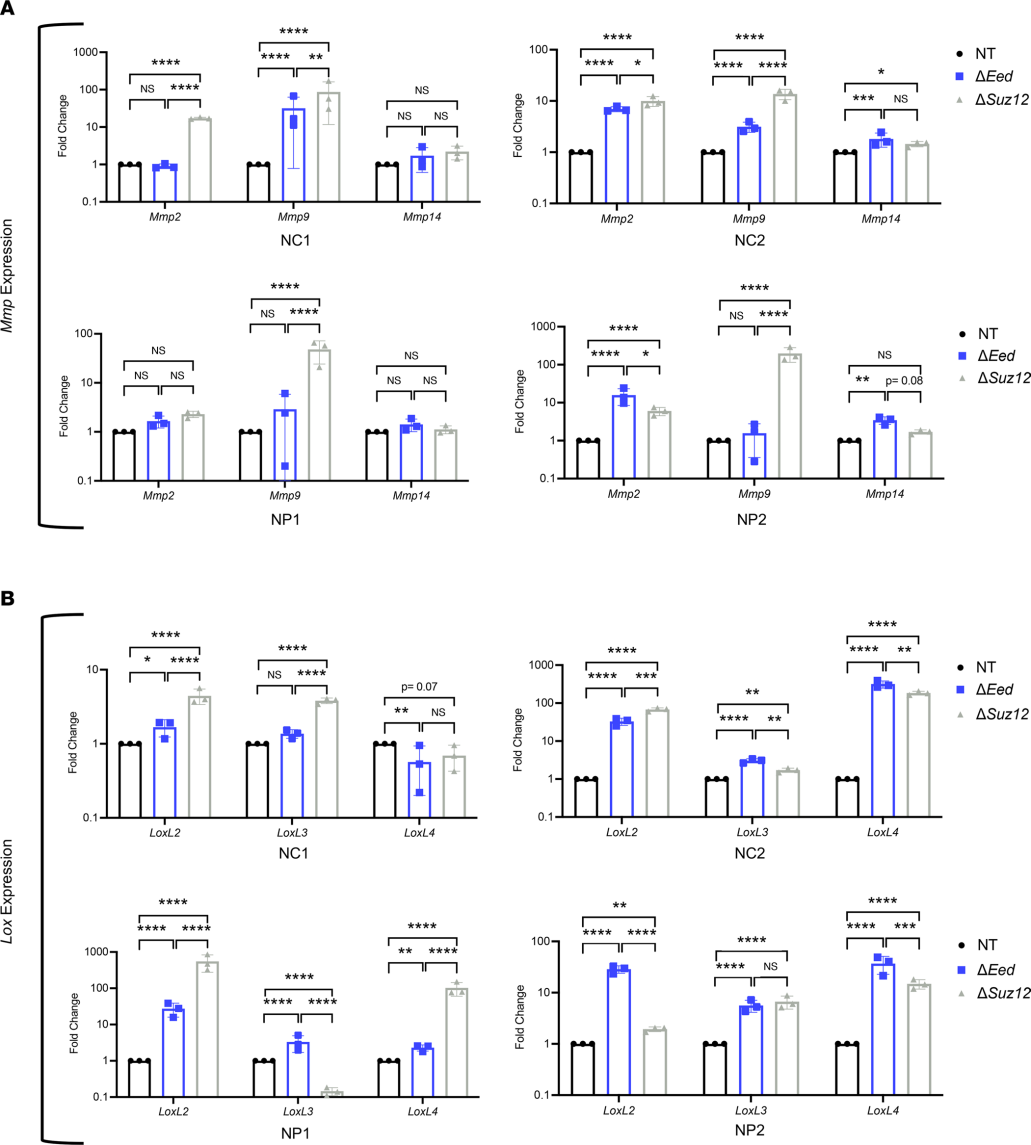
PRC2 deletion upregulates expression of *Mmp* and *Lox* enzyme families. (**A**) MMP enzyme gene expression by quantitative real-time PCR (qRT-PCR) in NP1, NP2, NC1, and NC2 isogenic cell lines shows upregulation of *Mmp9* and *Mmp2* with loss of either *Eed* or *Suz12*. Upregulation of *Mmp14* is seen in some cells with PRC2 loss (*n* = 3). (**B**) *Lox* enzyme gene expression by qRT-PCR in NP1, NP2, NC1, and NC2 isogenic cell lines shows upregulation of *Lox* family genes *LoxL2*, *LoxL3*, and *LoxL4* with loss of either *Eed* or *Suz12* (*n* = 3). qRT-PCR data analyzed by 1-way ANOVA with Tukey’s multiple comparisons. Data represent biological replicates with the mean ± SD; *P < 0.05, **P < 0.01, ***P < 0.001, ****P < 0.0001.

PRC2 controls a multitude of genetic targets, several of which have been linked to metastatic processes in other cancers. Since some of the metastasis-associated behaviors we observed centered on interactions with the surrounding matrix, we hypothesized that PRC2 loss may be altering expression of ECM-remodeling genes in MPNST cells. We focused on members of the MMP and LOX enzyme families, as these enzymes function together to remodel the matrix and maintain cellular homeostasis. Interactions between matrix-remodeling enzymes are a critical driver of metastatic potential, with recent studies identifying the importance of ECM enzyme profiles that regulate multiple players to drive metastatic phenotypes. We observed PRC2-regulated upregulation of MMP and LOX gene signatures across all 4 isogenic panels (Figure 4, A and B). In both NC cells, loss of either *Eed* or *Suz12* resulted in upregulation of *Mmp9* (Figure 4A). Induction of *Mmp2* was observed with *Suz12* loss in both NC1 and NC2 cells, while *Mmp2* was elevated in *Eed*-null NC2 cells. The protease *Mmp14* was elevated in NC2 cells with *Eed* and *Suz12* loss but was not altered in NC1 cells. Similar trends of MMP upregulation were seen in both NP isogenic series. Most notably, *Suz12* loss drove *Mmp9* expression in NP1 and NP2 cells, although *Eed* loss did not influence MMP9 levels. Expression of *Mmp2* and *Mmp14* was elevated in NP2 cells with PRC2 loss, and a trending increase of *Mmp2* was observed in NP1 cells.

We also examined expression of the LOX family members *LoxL2*, *LoxL3*, and *LoxL4*, which are involved in collagen remodeling. In all isogenic cell series, *LoxL2* was dramatically induced with *Eed* loss, while *Suz12* loss resulted in *LoxL2* induction in 3 of 4 cell panels (Figure 4B). Similarly, *LoxL4* was upregulated with PRC2 loss in both NC and NP cells. Levels of *LoxL3* were substantially higher in all 4 *Eed*-null cell lines, although only 2/4 isogenic series showed *LoxL3* induction following *Suz12* loss. While both Δ*Eed* and Δ*Suz12* cells had altered ECM gene signatures, we observed modest differences in profiles between cells with *Eed* or *Suz12* loss, suggesting some variations between PRC2 subunits and genetic backgrounds. However, the overall induction of ECM-remodeling enzyme signatures was consistently observed in each isogenic panel, suggesting this was a conserved PRC2-dependent event. Taken together, this analysis identifies strong PRC2-regulated induction of multiple *Mmp* and *Lox* transcripts in MPNST cells, particularly *Mmp9*, *LoxL2*, and *LoxL4*.

### LOX enzyme function is required for in vitro PRC2-regulated metastasis-associated phenotypes.

We next used pharmacological inhibitors to determine the roles of MMP and LOX activity on in vitro MPNST metastasis-associated phenotypes. Serum-starved cells were placed in the upper chamber of a collagen-coated Transwell and treated with the LOX family inhibitor β-aminopropionitrile (BAPN) or pan-MMP inhibitor GM6001 for 6 hours (Supplemental Figure 6A). Treatment of NC1 cells with BAPN was sufficient to inhibit invasion through collagen for Δ*Eed* and Δ*Suz12* cells, while there was no significant reduction of invasion in PRC2 wild-type cells. Similar PRC2-regulated inhibition of invasion was observed with BAPN treatment of NP1 cells (Supplemental Figure 6, B and D). Additionally, the LOX inhibitor decreased the formation of cell clusters in Δ*Eed* and Δ*Suz12* cells from both the NC1 and NP1 cell panels. BAPN treatment did not impact cell clustering in control cells with wild-type PRC2 (Supplemental Figure 6, B and D, and Supplemental Figure 7). These data suggest that LOX activity is a robust driver of metastasis in PRC2-deleted MPNSTs. In contrast to the LOX inhibitor, treatment with the pan-MMP inhibitor GM6001 impacted metastatic phenotypes independent of PRC2 status. In NP1 cells, GM6001 treatment decreased invasion in both PRC2 wild-type and ΔPRC2 cells (Supplemental Figure 6, C and E, and Supplemental Figure 7). MMP-dependent clustering was observed in NP1 Δ*Eed* cells, but not PRC2 wild-type or Δ*Suz12* cells. In NC1 cells, invasion was slowed in PRC2 wild-type and Δ*Suz12* cells with GM6001 treatment, while Δ*Eed* cell invasion was not affected. However, smaller clusters were observed only in GM6001-treated Δ*Eed* cells. Importantly, neither BAPN nor GM6001 treatment altered cell viability (Supplemental Figure 8).

**Figure 5:**
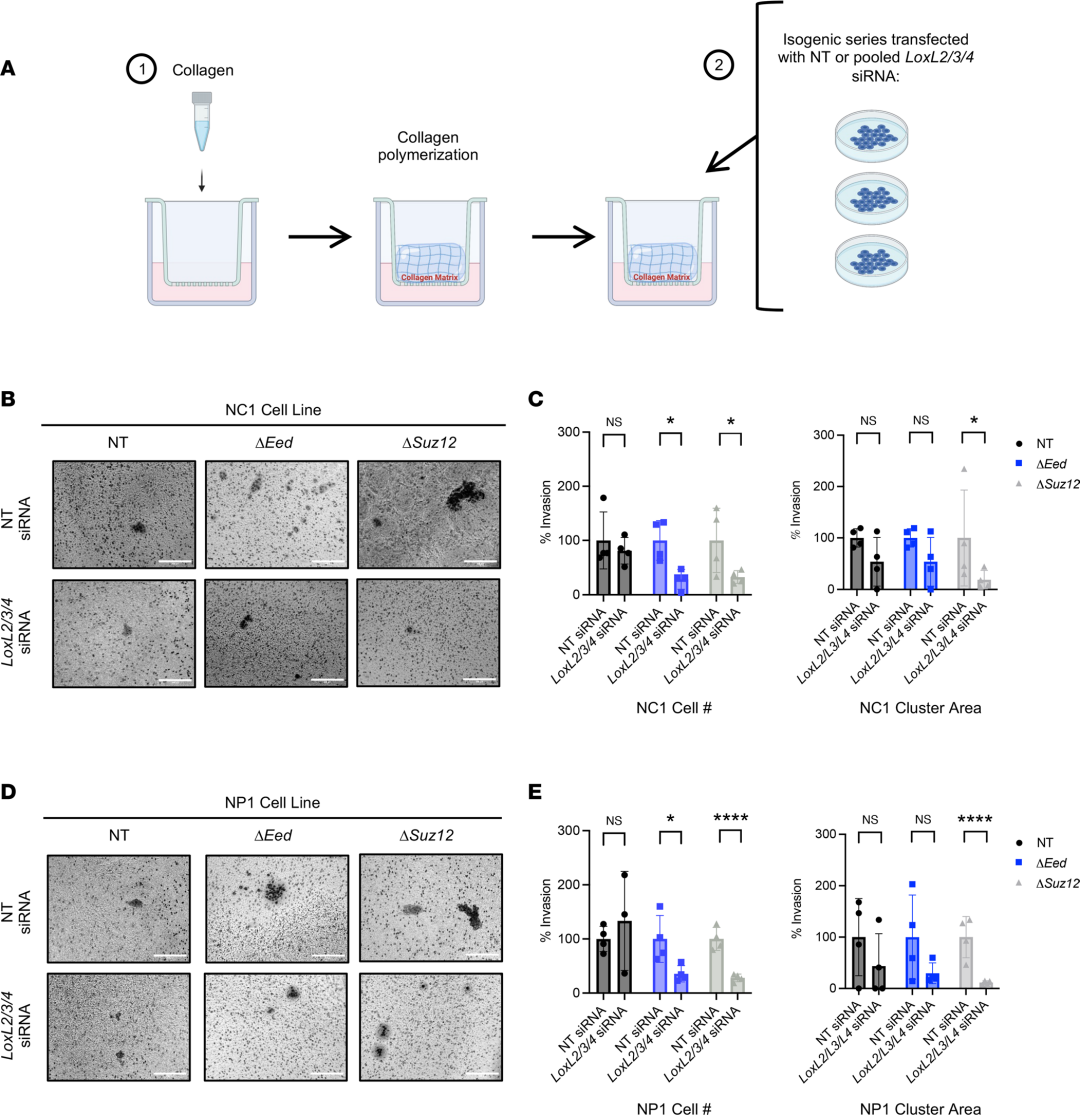
Inhibition of LOX enzyme function, but not MMP function, decreases PRC2-dependent metastatic phenotypes. (**A**) Schematic of Transwell invasion across collagen with either nontargeting control siRNA (NT siRNA) or pooled *Lox* siRNA transfection (*LoxL2/3/4* siRNA). (**B** and **D**) Representative images (original magnification, 20×) of cell Transwell invasion and clustering of NC1 (**B**) and NP1 (**D**) isogenic cell lines 48 hours posttransfection (*n* = 4). (**C** and **E**) Transwell invasion cell number (left) and cluster area (right) with either NT siRNA or *LoxL2/3/4* siRNA 48 hours after transfection for NC1 (**C**) and NP1 (**E**) isogenic cell lines (*n* = 4). Transwell invasion assays show increased invasion through collagen of Δ*Eed* and Δ*Suz12* cells compared with nontargeting control by Kruskal-Wallis with multiple comparisons. Data represent biological replicates with the mean ± SD; *P < 0.05, **P < 0.01, ***P < 0.001, ****P < 0.0001.

To further explore the role of *Lox* targets in MPNST cell migration, we performed siRNA knockdown studies (Figure 5). First, we tested the impact of knocking down multiple *Lox* family members in tandem, choosing targets with the highest increase upon PRC2 loss: *LoxL2*, *LoxL3*, and *LoxL4*. Similar to BAPN treatment, there was no impact on invasion in PRC2 wild-type NC1 and NP1 cells treated with pooled *LoxL2/3/4* siRNAs. However, invasion was significantly impaired in cells with PRC2 loss, supporting our previous inhibitor data (Figure 5, B–E, and Supplemental Figure 9). Cluster area was greatly decreased in pooled *Lox* siRNA–treated Δ*Suz12* cells, but the impact on Δ*Eed* cells was less pronounced (Supplemental Figure 10 A and E). Next, we examined the role of individual *Lox* targets. In NP1 cells, single targeting of *LoxL2*, *LoxL3*, or *LoxL4* resulted in a greater than 50% decrease of invasion in both Δ*Suz12* and Δ*Eed* cells (Supplemental Figure 10, B–D). However, combination of all 3 targets decreased invasion by greater than 90%, suggesting LOX members play distinct and complementary roles in MPNST metastasis-associated phenotypes. Similar results were observed in NC1 cells (Supplemental Figure 10, F–H). In both cell lines, cluster area was decreased in Δ*Suz12* cells treated with any *LoxL* siRNA. Taken together, these data identify a strong role for LOX family enzymes in PRC2-regulated invasion and cell clustering, while MMP enzymes may be involved in more subtle cell clustering phenotypes, particularly in *Eed*-null cells.

### PRC2 deletion remodels the ECM and drives metastasis in vivo.

**Figure 6:**
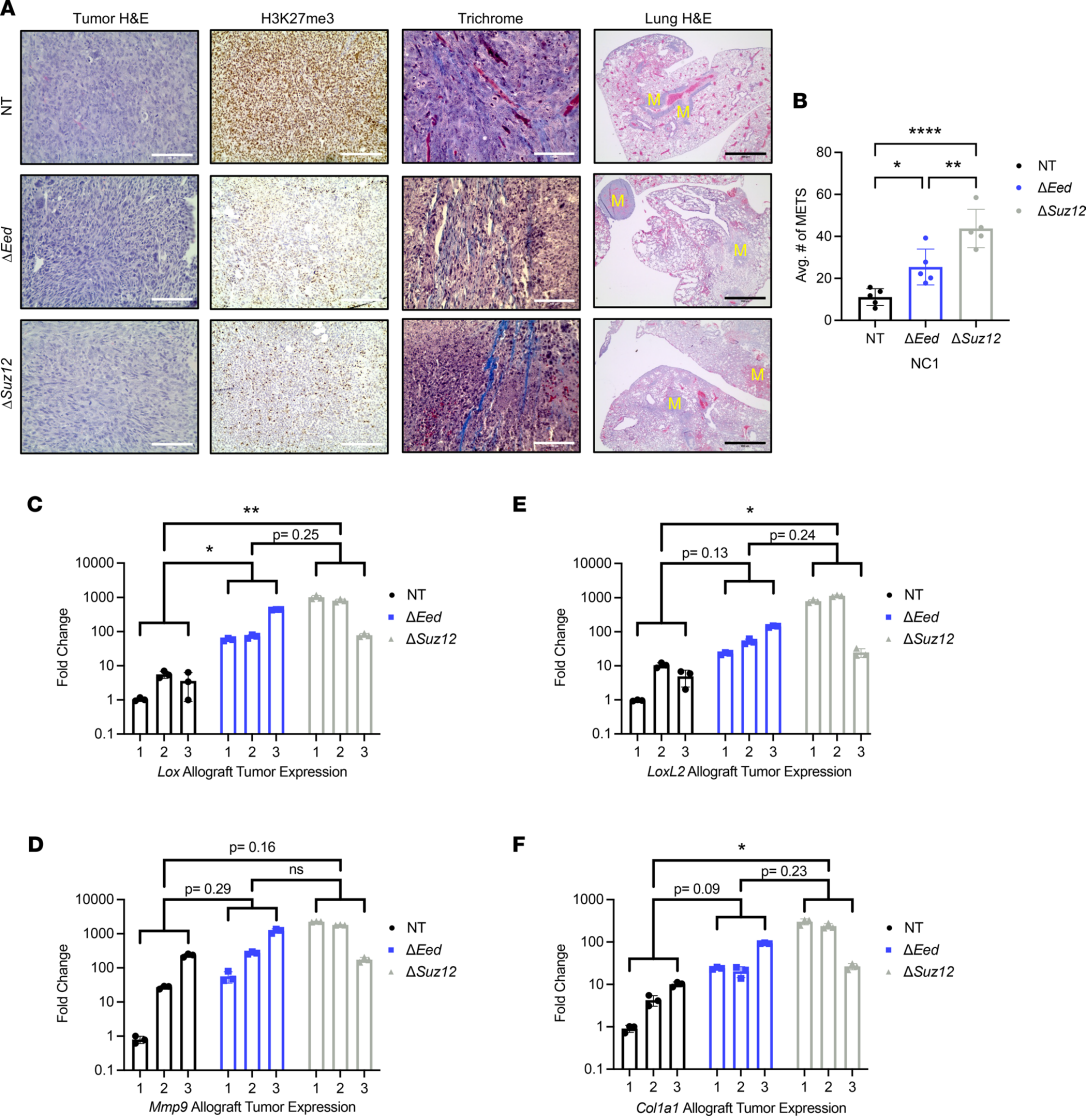
PRC2 loss increases lung metastasis, tumor fibrosis, and expression of *Mmp* and *Lox* enzyme families in vivo. (**A**) Orthotopic tumors generated from the NC1 isogenic series show characteristic MPNST pathology is maintained with PRC2 loss (H&E, left panels). Δ*Eed* and Δ*Suz12* tumors maintain low H3K27me3 staining compared with NT tumors (IHC, middle panels). Representative histological lung sections show increased metastases with PRC2 loss (H&E, right panels; metastases indicated by yellow “M”). (**B**) Increased metastatic lung area in mice with Δ*Eed* (*n* = 5) and Δ*Suz12* (*n* = 5) tumors compared with NT (*n* = 5). Metastatic (MET) area quantification was performed on 5 lungs per group. Histological sections were taken at 0 μm, 100 μm, 200 μm, and 300 μm depths, and 4 images per section were taken at 4× objective. Data were analyzed using 1-way ANOVA with Tukey’s multiple comparisons. (**C**–**F**) Gene expression analysis of whole-tumor lysate shows upregulation of *Lox*, *LoxL2*, *Mmp9*, and *Col1a1* mRNA in Δ*Eed* (*n* = 3) and Δ*Suz12* (*n* = 3) tumors compared with NT. Three individual tumors per genotype are shown; expression relative to a single NT tumor. Data analyzed using 1-way ANOVA with Tukey’s multiple comparisons. Scale bars at 200 μm. Data represent biological replicates with the mean ± SD; *P < 0.05, **P < 0.01, ****P < 0.0001.

To identify the effects of PRC2 loss in orthotopic tumors, we implanted the NC1 isogenic series into the sciatic nerves of NSG mice. MPNSTs generated from PRC2-deleted cells displayed histologically similar pathology to nontargeting controls (Figure 6A). IHC demonstrated that loss of H3K27me3 was maintained in PRC2-deleted MPNSTs. Upon gross analysis during terminal tumor harvest, we observed that PRC2-deleted tumors were more rigid and appeared to have a firmer and denser texture. Staining with Masson’s trichrome revealed high fibrosis throughout the PRC2-null tumors, including the formation of long collagen cables and larger collagen nests (Figure 6A). Other groups have also reported elevated tumor fibrosis following Eed knockout in non–small cell lung cancer mouse models (27). We also observed a significant increase in lung metastases from mice with PRC2-deleted tumors, consistent with tail vein data shown in Figure 3 (Figure 6, A and B). These orthotopic in vivo data further support a robust PRC2-regulated metastatic phenotype. We next performed qRT-PCR on whole-tumor lysates to examine the in vivo expression of key matrix-remodeling enzymes. Similar to data with cultured cell lines, we observed elevated levels of *Lox*, *LoxL2*, and *Mmp9* mRNA in ΔPRC2 tumors (Figure 6, C–F). Additionally, the fibrosis phenotype prompted us to examine gene expression of structural ECM components, and we identified elevation of *Col1a1* mRNA in PRC2-deleted allografts (Figure 6F). When combined with in vitro gene expression, pharmacological inhibitor studies, and siRNA experiments (Figures 4 and 5 and Supplemental Figure 6), these data suggest that PRC2 loss alters expression of key ECM-remodeling factors in MPNST that are associated with increased tumor metastasis.

### Loss of PRC2 in patient samples correlates with metastasis and increased fibrosis.

**Figure 7:**
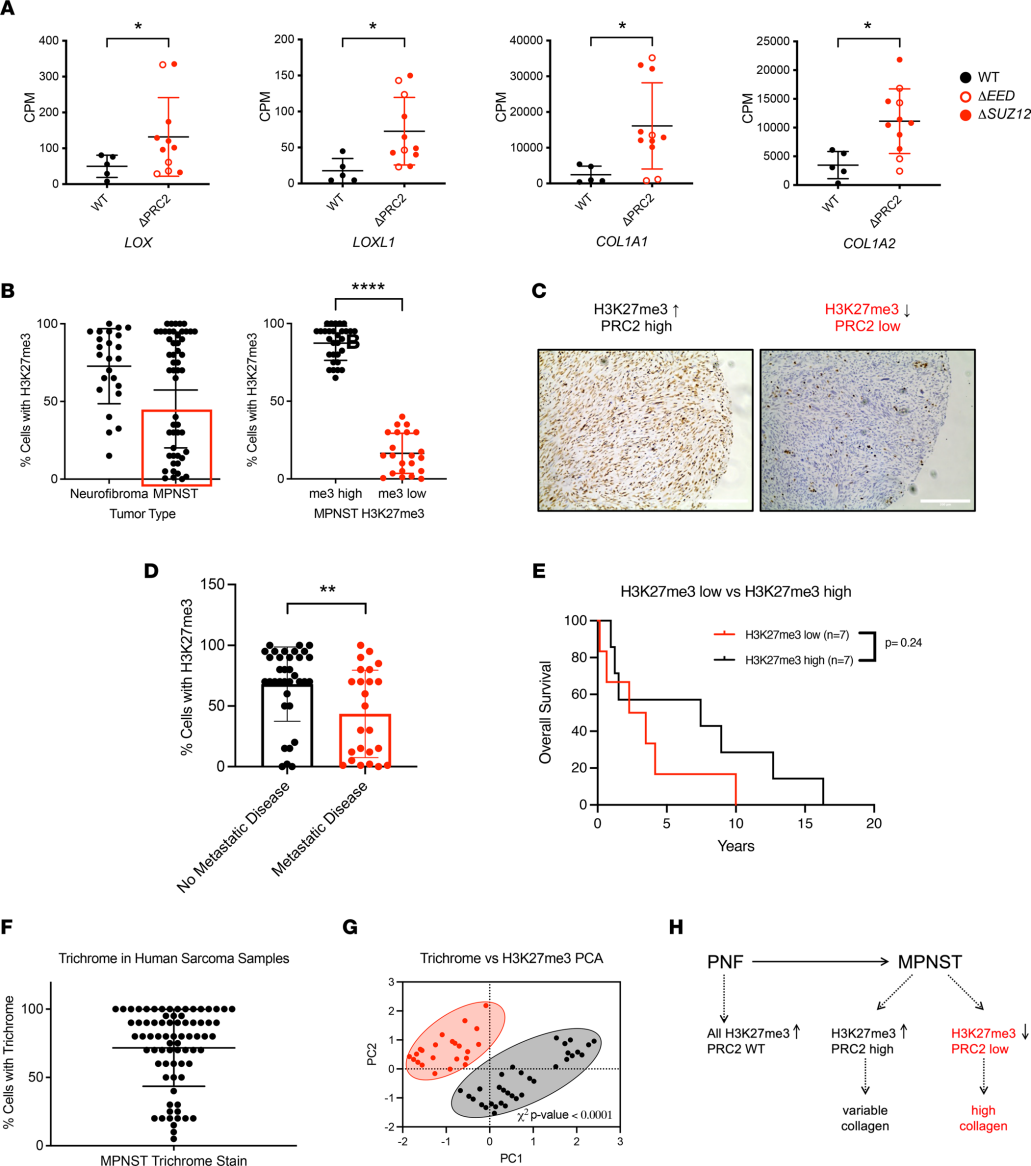
PRC2 loss correlates with increased metastasis, fibrosis, and ECM gene expression in patient MPNST. (**A**) Expression of *LOX*, *LOXL1*, *COL1A1*, and *COL1A2* mRNA is increased in PRC2 mutant (ΔPRC2) compared with PRC2 wild-type (WT) patient samples. Previously published data set with Fisher’s exact *t* test (19). (**B**–**G**) Analysis of a tissue microarray and correlative clinical data. (**B**) IHC staining for H3K27me3 identifies PRC2-high and PRC2-low MPNSTs (red box) in tumor cores. (**C**) Representative images of MPNSTs with high (left) and low H3K27me3 (right). (**D**) Metastatic disease correlates with lower H3K27me3 in MPNST patients by Fisher’s exact *t* test. (**E**) Overall survival is decreased in patients with lower H3K27me3 scores. Classification of H3K27me3-low and -high indicated by red box in **B** (*n* = 14). (**F**) Histological analysis of Masson’s trichrome staining in MPNST tumor cores shows a broad range of tumor fibrosis. (**G** and **H**) PRC2 loss correlates with high trichrome staining and increased tumor fibrosis. Principal component analysis (PCA) and χ^2^ analysis of H3K27me3 and Masson’s trichrome percentage cell-positive staining with low and high classifiers assigned as indicated by a red circle (H3K27me high) and black circle (H3K27me3 low). Data represent biological replicates with the mean ± SD; *P < 0.05, **P < 0.01, ****P < 0.0001. CPM, counts per million.

Patient samples of MPNST are incredibly limited, with some of the largest gene expression data sets containing fewer than 30 samples (18, 19, 21). We examined a previously published RNA-Seq data set containing 5 PRC2 wild-type and 11 PRC2-mutant MPNSTs to evaluate expression of genes involved in ECM-remodeling enzymes and matrix production (19). Strikingly, PRC2-mutant tumors displayed strong enrichment of *LOX*, *LOXL1*, *COL1A1*, and *COL1A2* transcripts compared with PRC2 wild-type MPNSTs (Figure 7A). Since the paucity of larger data sets makes clinical correlative studies difficult, we examined tissue microarrays (TMAs) of patient-matched neurofibroma (*n* = 24) and MPNST (*n* = 26) pairs, in addition to 10 additional MPNST samples. We stained the TMAs for H3K27me3, a well-accepted clinical marker of PRC2 loss (41). As expected, H3K27me3 staining remained high in neurofibromas, which have intact PRC2 (Figure 7, B and C). H3K27me3 staining in MPNSTs showed a strong bimodal distribution (Figure 7C), with either high positivity (>60% cells) or low positivity (<30% cells). We used these natural cutoffs to categorize samples into PRC2-high and PRC2-low groups. We next examined clinical correlates of patients with high versus low H3K27me3 staining. Patients who developed metastatic disease had lower levels of H3K27me3 staining, suggesting a strong correlation between PRC2 loss and increased metastasis (Figure 7D). Additionally, overall survival was decreased in patients with low H3K27me3, although this was not significant, possibly due to the small number of patients available for follow-up (Figure 7E). We next examined if tumor fibrosis correlated with PRC2 loss, as suggested by our preclinical data. The TMAs were stained with trichrome, and percentage of positive cells was quantified by a sarcoma pathologist. Samples in the top 50% of trichrome intensity displayed the lowest H3K27me3 levels, identifying a strong correlation between PRC2 loss and increased fibrosis in patient samples (Figure 7, F–H). As shown in the diagram (Figure 7H), while we observed that PRC2 wild-type MPNSTs had variable levels of collagen, PRC2-deleted MPNSTs all had high levels of collagen. Of note, it is possible that analysis of whole-tumor mounts may show a more pronounced fibrotic phenotype, as tissue selection for TMA analysis is biased toward sections with high neoplastic cell content that may artificially underrepresent the fibrotic density of the tumors. These data demonstrate a strong correlation between PRC2 loss and development of metastatic disease, increased fibrosis, and decreased survival in patients with MPNSTs.

## Discussion

Metastasis is a leading cause of death for patients with MPNSTs, but the molecular mechanisms driving this event are poorly understood. In this study, we tested the hypothesis that frequently occurring loss-of-function mutations in the histone methyltransferase PRC2 can drive MPNST metastasis. Following deletion of *Eed* or *Suz12* in MPNST cells, we observed PRC2-regulated metastasis-associated phenotypes, including increased invasion through collagen, elevated formation of cellular clusters, and enlarged lung metastases following tail vein injection. PRC2 loss resulted in upregulation of key ECM remodeling enzymes in all 4 isogenic cell panels, particularly *Mmp9*, *LoxL2*, and *LoxL4*. Pharmacological inhibition and genetic knockdown of the LOX enzyme family slowed migration in a PRC2-dependent manner, suggesting a role in metastatic dissemination. Furthermore, PRC2 loss in orthotopic allografts induced gene expression of *Lox* and *Mmp* targets while increasing fibrosis and lung metastasis. Finally, examination of human samples determined that PRC2 loss correlated with increased tumor fibrosis, metastatic outcome, and upregulation of matrix-remodeling enzymes. Thus, this study demonstrates that the PRC2 pathway is a critical driver of matrix remodeling and metastasis in MPNST that is associated with upregulation of ECM-modifying enzymes. These findings could have broad implications for clinical management of MPNST by using PRC2 status and/or ECM features to identify patients at risk for metastasis.

Several distinct mechanisms could be responsible for PRC2-mediated control of MPNST metastasis and fibrosis. First, as explored in this study, PRC2 loss can induce expression of *Mmp* and *Lox* enzymes, resulting in remodeling of the ECM to facilitate local invasion of tumor cells into the surrounding stroma. A direct role for PRC2 control of ECM-remodeling enzymes has been reported in several cancers, as PRC2 can induce epigenetic silencing through binding to *MMP* promoters (29). This canonical H3K27me3-associated transcriptional repression of ECM targets also occurs through regulation of the tissue inhibitors metalloproteinase family (TIMPs), negative enzymatic regulators of MMPs. In breast cancers, where PRC2 activity is elevated in metastatic disease, high levels of enhancer of zeste 2 polycomb repressive complex 2 (EZH2) are associated with repression of *TIMP* expression via H3K27me3 silencing (42). This results in increased MMP activity, driving metastatic phenotypes in these breast cancer models. Similar repression of TIMPs via EZH2 overexpression has been observed in ovarian and prostate cancers (43, 44). Second, PRC2-regulated targets may be driving additional parts of the metastatic cascade in MPNST. For example, PRC2 has been linked to expression of profibrotic cytokines, such as TGF-β or IL-6. These promigratory factors could play important roles in the reciprocal crosstalk between fibroblasts and tumor cells, which is important for tumor progression, cell migration, and ECM remodeling. The role of PRC2 in TGF-β activation can occur either through direct regulation or through an intermediary target, such as miRNA-490 in patients with glioblastoma (45). Indeed, PRC2 represses hundreds of cytokines and cytokine receptors, and this targeting is elevated in cancer cells compared with noncancer cells (46). Third, the function of PRC2 in metastasis could be independent of histone methyltransferase activity, as seen in several other cancers. For example, in metastatic breast cancer, cytoplasmic binding of phosphorylated EZH2 directs regulation of cytoskeletal elements responsible for cell migration and invasion (5). Similarly, in metastatic bone lesions, EZH2 functions as a transcriptional cofactor for RNA polymerase II, resulting in increased integrin β1 signaling and subsequent downstream activation of TGF-β (47).

We observed that PRC2-deleted cells migrating through collagen had increased cellular clustering compared with wild-type controls. Although the implications of this clustering phenotype are not clear, we have reported these observations to contribute to conversations about metastasis-associated phenotypes in mesenchymal cells. Indeed, many sarcoma cells have intrinsic invasion and migration properties based on their mesenchymal nature, and caution is warranted when using traditional assays that are common for epithelial biology. Indeed, collective migration and various clustering phenotypes have been documented in epithelial cancers such as breast and colon cancer (48). Although studies have demonstrated that epithelial collective migration is dependent on stem cell–like gene programs and complex cadherin biology, data from our MPNST cells show that PRC2-regulated clusters have minimal changes in *Sox2* expression (data not shown). This suggests that the predominating mechanism of PRC2-regulated clustering in MPNSTs is driven by manipulating the ECM rather than altering stem-like properties of these mesenchymal tumors.

Limited patient data make it difficult to determine if *EED* and *SUZ12* play functionally distinct or overlapping roles in PRC2-regulated pathogenesis of MPNSTs. One goal of this study was to gain a better understanding of subunit-specific roles in PRC2 pathogenesis. We observed moderate differences in migration phenotypes, inhibitor sensitivity, and gene expression profiles among cells with loss of different PRC2 subunits, suggesting that PRC2-mediated control of metastasis-associated phenotypes is complex and involves regulation of multiple signaling pathways in a context-specific manner. In our analysis of multiple pairs of isogenic cell panels, we observed that although overall metastasis-associated features were similar between Δ*Eed* and Δ*Suz12* cells, several phenotypes were differentially affected, such as clustering and migration speed. For example, in the time-lapse movie analysis, total displacement was similarly increased in Δ*Eed* and Δ*Suz12* cells, while higher migration speed and net distance traveled were observed only in Δ*Eed* cells compared with wild-type controls. We also observed selective impact on *Mmp* and *Lox* enzyme regulation between *Eed* and *Suz12* deletion, suggesting that different ECM profiles may be responsible for metastatic events within each genetic context. Most notably, *Mmp9* was consistently upregulated with loss of either *Eed* or *Suz12* loss in NC cells. However, in NP cells only *Suz12* loss was able to induce *Mmp9* expression, while *Eed* loss did not impact *Mmp9* levels. These differences translate into functional assays, as treatment with the MMP inhibitor GM6001 was sufficient to decrease overall invasion in both Δ*Eed* and Δ*Suz12* cells, but smaller clusters were observed only in GM6001-treated Δ*Eed* cells. Fewer subunit-dependent phenotypes were seen in analysis of the LOX family. Upregulation of *LoxL2* and *LoxL4* occurred following loss of either *Eed* or *Suz12*, while *LoxL3* was more consistently induced by *Eed* loss (4/4 cell lines) than *Suz12* loss (2/4 cell lines). Similarly, BAPN treatment inhibited invasion and clustering phenotypes in both Δ*Eed* and Δ*Suz12* cells. These subunit-specific effects on distinct metastasis-associated processes speak to different types of multicellular dynamics that impact collective migration. While each individual phenotype may be controlled by slightly different mechanisms, we suggest there is an additive impact on overall cellular migration and invasion that combines to result in greater metastatic potential. Importantly, while several in vitro metastatic phenotypes occurred more frequently with *Eed* loss, both Δ*Eed* and Δ*Suz12* cells produced similar levels of lung metastases in tail vein and allograft studies, underscoring the importance of both subunits in driving MPNST metastasis.

PRC2-regulated tumor fibrosis may have implications for drug delivery in patients. Notably, clinical management of MPNST frequently involves systemic treatment with doxorubicin as a first-line agent. However, collagen-dense stroma can impede drug delivery, as increased interstitial fluid pressure and physical barriers can prevent therapeutic agents such as doxorubicin from entering the tumor (49, 50). Thus, there are strong translational implications for increased fibrosis in PRC2-deleted MPNST. For example, if drug delivery is limited by elevated matrix deposition, this suggests PRC2-deleted MPNST may benefit from stromal reengineering therapy with antifibrotic agents. Indeed, several groups are examining treatment with hyaluronic acid–targeted therapies to increase delivery of chemotherapies into MPNSTs (51). Importantly, while our data identify altered ECM biology in PRC2-deleted tumors, we have not yet uncovered the mechanism driving this fibrotic remodeling. Instead of neoplastic tumor cells, the source of increased fibrosis could be from surrounding stroma, such as MPNST-resident fibroblasts. Thus, targeting profibrotic cytokines may interrupt the crosstalk between tumor cells and cancer-associated fibroblasts that contribute to matrix production.

Up to 50% of MPNSTs metastasize, and an improved understanding of the metastatic process will allow physicians to derive potential biomarkers of metastatic disease and ultimately target these pathways prior to metastatic dissemination. Due to the large number of cancers with overactive PRC2 signaling, current efforts are focused on developing small molecule inhibitors of EZH2 activity. Therefore, MPNST-focused approaches would be better suited to focus on druggable downstream targets of PRC2 loss, such as MMP or LOX enzyme activities. In animal models, pharmacological inhibitors of ECM-remodeling enzymes can suppress metastatic growth when given early in the metastatic process (52, 53). However, the clinical use of these agents has been hindered by poor outcomes and high toxicity in cancer patients (22–24). Early clinical trials with broad-spectrum inhibitors failed to improve survival, though these studies were conducted on patients with advanced disease. Recent success with more selective FDA-approved MMP inhibitors is showing improved pharmacokinetics and less toxicity than previous formulations (22). New pan-LOX and LOXL2 inhibitors have reached phase I/II clinical trials for fibrotic diseases, and studies report these agents are well tolerated with excellent safety profiles (54). The pan-LOX inhibitor PXS-5505 was granted orphan drug designation for myelofibrosis in mid-2020, and trials with this agent have begun in several cancers, including liver and blood; results are not yet available (ClinicalTrials.gov NCT04676529, NCT05109052). The identification of PRC2-regulated metastatic pathways identified in this study provides strong preclinical rationale for developing therapies targeting ECM-remodeling enzymes and tumor fibrosis to improve outcomes in patients with MPNST.

## Methods

### Generation of isogenic PRC2-deleted cell series and cell proliferation assays.

Published murine MPNST cells (see refs. 30, 32, 34) were transfected with the px459 plasmid (Addgene 62988) expressing Cas9 and a single guide RNA against *Suz12* (Δ*Suz12*), *Eed* (Δ*Eed*), or a nontargeting control (tdTomato). (55) Cells were grown in DMEM (Thermo Fisher Scientific, 11965092) supplemented with 10% fetal bovine serum, 1% sodium pyruvate (Gibco, 11360-070), and 1% penicillin/streptomycin (pen/strep) (Gibco, 15140-122). Clonal cell lines were isolated by limiting dilution, and *Suz12* and *Eed* indels were verified by sequencing followed by ICE analysis (Synthego, https://ice.synthego.com/#/). Western blot confirmed loss of EED (Cell Signaling Technology, EED 51673S), SUZ12 (Cell Signaling Technology, SUZ12 D39F6, 3737S), and H3K27me3 (Cell Signaling Technology, H3K27me3 C36B11, 9733S) in PRC2-deleted cell lines. For cell proliferation assays, 100 μL of resuspended cells (3.367 × 10^5^ cells/mL) were plated into 96 wells in triplicate. Cell number was determined 1, 3, 4, and 5 days postseeding by adding 100 μL of fresh media and 20 μL of alamar blue (MilliporeSigma, R7017) for 2 hours and reading fluorescence at 590 nm (BioTek Synergy HT).

### In vitro metastatic phenotype studies.

For Transwell assays, cells were cultured in DMEM supplemented with 10% fetal bovine serum, 1% sodium pyruvate, and 1% pen/strep. Cells were maintained at 37°C with 5% CO_2_ in a humidified cell culture incubator. Cell lines were grown to 80%–90% confluence, trypsinized using 0.25% EDTA Trypsin (Gibco, 25200-056), counted, spun down at 500*g* for 5 minutes, and resuspended in serum-free media (1.0 × 10^6^ cells/mL). For migration studies, 100 μL of cells were placed in the Transwell insert of 24-well plates (Corning, CLS3422), placed over wells with fully supplemented media, and moved to the incubator for 4–6 hours. Transwells were stained with hematoxylin (Vector Laboratories, H-3401) for cell counting. For invasion assays, Rat Tail Collagen 1 (Corning, 354236) was prepared at 0.8 mg/mL by sequentially adding 200 μL of 4× DMEM, 10.4 μL 1 M NaOH, 1.342 mL double-distilled water (ddH_2_O), and 448 μL of collagen. The 40 μL of diluted collagen was placed on a prechilled Transwell insert and allowed to polymerize in a humidified cell culture incubator at 37°C with 5% CO_2_ for 30 minutes. Following collagen polymerization, cells were resuspended and added to Transwell inserts as described above. Inhibitor studies used either 50 μM GM6001 (MilliporeSigma, 364206) or 400 μM BAPN (MilliporeSigma, A3134). For viability assays with BAPN and GM6001, 100 μL of NP1 and NC1 isogenic series (1.0 × 10^6^ cells/mL) were plated in 12-well plates (CellStar, 665180) with either media alone, GM6001 (50 μM), BAPN (400 μM), or gemcitabine (30 μM) (MedChemExpress, HY-B0003) for 6 hours or 24 hours. Cell viability was assessed via trypan blue (Gibco, 15250-061) exclusion. For cell counting, cells remaining in the top of the insert were removed with a cotton swab, and the insert was transferred to a fresh plate to fix in 600 μL of 70% ethanol for 10 minutes. Next, inserts were stained in 600 μL of hematoxylin for 10 minutes, rinsed in ddH_2_O, and dried overnight. Inserts were imaged at 20× original magnification, with an EVOS light imaging system to count the number of cells. Additional clustering analysis was completed using ImageJ software (NIH).

### 3D collagen movies.

A 35 mm tissue culture-treated plate (Falcon, Corning, 353001) was coated in collagen as above. Resuspended cells were placed on the collagen matrix and incubated at 37°C with 5% CO_2_ for about 18 hours. Cultures were then moved to a stage incubator (20/20 Technology) providing a humidified 5% CO_2_, 37°C atmosphere on a Leica DMIRE2 inverted microscope. OpenLab software (Improvision) running on an Apple iMac computer controlled illumination and image acquisition. Images were captured using a 10× phase-contrast objective every 5 minutes for 20 hours. Total distance traveled and migration speed were calculated using MtrackJ ImageJ plug-in software (56). A total of 40 cells per genotype were tracked at 40-minute intervals across the 20-hour video. ImageJ was used to assemble QuickTime movies with a playback rate of 12 frames/s (3,600-fold real time).

### qRT-PCR.

For cell lines, RNA was collected in 1 mL of Trizol (15596018, Thermo Fisher Scientific) and stored at –80°C. RNA was extracted using Direct-zol RNA MiniPrep (R2052, Zymo Research) per the manufacturer’s instructions with additional DNA digestion step. Tumor tissue RNA was collected by storing terminal tumor tissue in RNA*later* (AM7020, Thermo Fisher Scientific) at –20°C. Tumors (*n* = 3 per genotype) were homogenized using a mortar and pestle in liquid nitrogen and resuspended in Trizol. cDNA was synthesized from 1 μg of RNA (for both cell lines and whole tumor) using iScript (1708891, Bio-Rad). qRT-PCR was performed using PowerUp SYBR Green 2x Master Mix (A25778, Thermo Fisher Scientific) per the manufacturer’s instructions on an Applied Biosystems 7900HT instrument using the ΔΔC_t_ relative to *B2m* (cell lines) or *18s* expression (whole tumor) (Genomics Division of the Iowa Institute of Human Genetics, University of Iowa).

### siRNA metastatic phenotype studies.

Cells were maintained as previously stated. For siRNA studies, transfection efficiency was tested in NP1 and NC1 isogenic series by plating 2,000 and 4,000 cells in 96-well plates (CellStar, 655180). Transfection efficiency was assessed via flow cytometry following transfection of cells with TYE-563 fluor (IDT, 51-01-20-19) using Jet PRIME transfection kit per manufacturer’s instructions (101000046). All siRNA constructs were selected from the IDT website. For transfection experiments, cells were transfected with either a nontargeting control construct (50 nM) or *LoxL2/3/4* constructs (single, 50 nM; pooled, 10 nM/construct) per manufacturer’s instructions scaled up for a 6-well (CellStar, 657160) format. Forty-eight hours posttransfection transcript expression was analyzed via qRT-PCR (see *qRT-PCR*). Transwell experiments were conducted and analyzed as previously stated 48 hours posttransfection.

### Mouse studies.

NSG mice (The Jackson Laboratory) were maintained at the University of Iowa. Male and female mice over 7 weeks old were used for all studies. All cells were about 90% confluent on the day of injection. Cells were trypsinized, washed, and resuspended in sterile PBS containing calcium chloride and magnesium chloride (Gibco, 14040-133). For tail vein injections, mice were injected with 1 × 10^6^ cells in the tail vein (*n* = 10 mice per cell genotype). Mice were monitored for signs of respiratory distress, including labored breathing, hunched posture, and ruffled fur. Lungs were inflated with 10% formalin and processed for histology. For orthotopic allograft tumors, mice were injected with 2.5 × 10^3^ NC1 cells in the sciatic nerve as previously described (33). Tumors were harvested when they reached 1,500 mm^3^ or when animals showed signs of distress. At sacrifice, primary tumor tissue was collected for molecular analysis or histology. For CFSE tail vein injection studies, cells were maintained as previously stated. For CFSE studies, the CFSE staining protocol was optimized by testing varying combinations of cell concentrations (1.0 × 10^6^ and 4.0 × 10^6^ cells/mL) and CFSE dye (0.5, 1.0, 2.0 μL of CFSE stock, 5 μM) per the manufacturer’s instructions (BioLegend, 423801). Retention of CFSE stain was tested 0, 24, 48, and 72 hours poststaining at optimal staining parameters (1.0 × 10^6^ cells/mL, 1.0 μL of CFSE stock) in the NC1 isogenic series via flow cytometry. Cell viability and CFSE toxicity were assessed at all time points by trypan blue (Gibco, 15250-061) exclusion and ZombieAqua stain (BioLegend, 423101). For CFSE-labeled tail vein injections, cells were stained using optimal staining parameters. Then, 24 hours poststaining, cells were prepared for tail vein injections (see *Mouse studies*). Next, lungs were harvested 24 hours after tail vein injection, digested as previously described (STEMCELL Technologies, PR00044), and analyzed via flow cytometry.

### Histological analysis.

Terminal tumor tissue and whole lungs were stored in 10% neutral buffered formalin for fixation. Formalin-fixed, paraffin-embedded tissues were sectioned and stained with hematoxylin (Vector Laboratories, H-3401) and eosin (Harleco, 200-12) to evaluate tissue morphology. Trichrome staining was performed at the University of Iowa Comparative Pathology Laboratory. For IHC, sections were deparaffinized in xylene and rehydrated through a series of ethanol washes. Antigen retrieval was performed in citrate-based buffer (Vector Laboratories). After washing, samples were stained using VECTASTAIN Elite ABC-HRP kit, per the manufacturer’s instructions (Vector Laboratories). Antibodies included H3K27me3 (Cell Signaling Technology; H3K27me3 clones C36B11, 9733S, used 1:200). Slides were counterstained using IHC-optimized hematoxylin (Vector Laboratories) and mounted with Permount (Fisher Chemical, SP15-500). Lung metastases were quantified from H&E-stained lung sections by evaluating 4 independent sections at 100 μm intervals through the lung. Images were taken using an EVOS light imaging system at 10×, 20×, or 40× original magnification, with figure scale bars at 200 μm.

### Human MPNST gene expression.

Patient MPNST gene expression data were collected from a published data set of 16 MPNSTs (phs000792.v1.p1) (19). Raw fastq files were derived from SRA files, and reads were mapped to the human genome (hg19) with STAR (57) before quantification with featureCounts (58). Sample quality was assessed by calculating the proportion of assigned, unassigned, and multimapped reads in each sample. SRR1577703 was excluded from the analyses due to over 50% of the sample having multimapped reads. Finally, count data were normalized into counts per million with edgeR (59), and expression was compared based on PRC2 status for select genes.

### Clinical patient sample analysis.

TMAs were generated by pathologists. Thirteen paired (from the same patient) neurofibromas and MPNSTs were identified, and an additional 12 unpaired MPNSTs were obtained from the University of Iowa Department of Pathology with previous approval from the Institutional Review Board (IRB 201408808). TMAs were constructed by arraying the tumors in duplicate (28 cases) or triplicate (10 cases). We took 1.0 mm cores from formalin-fixed, paraffin-embedded tissue and assembled them using an MTA-1 tissue arrayer from Beecher Instruments. TMAs were stained with Masson’s trichrome and H3K27me3 as previously noted and evaluated for staining intensity (0–3+) and extent (0%–100%) with an H-score (intensity × percentage) calculated. Extent of trichrome staining was evaluated as the percentage of ECM space demonstrating staining. Medical record review for each patient was performed to identify development of metastases, duration of clinical follow-up, and vital status.

### Statistics.

Statistical analysis was performed using the Prism 9 software (GraphPad), and a *P* value less than 0.05 was considered statistically significant. Kaplan-Meier survival curves were analyzed by Gehan-Breslow-Wilcoxon test. Analysis of in vitro growth, Transwell migration, 3D collagen movie quantification, and qRT-PCR and metastatic lung area was performed by 1-way ANOVA with Tukey’s multiple-comparison test with Bonferroni’s adjustment. Analysis of Transwell invasion studies was performed by Kruskal-Wallis test with Dunn’s multiple comparisons test. Cell clustering analyses were analyzed by Poisson’s regression. Analysis of Transwell drug studies was performed by either unpaired Welch’s *t* test (number of cells) or β-regression (percentage area) within each genotype. Analyses of human gene expression and clinical metastatic outcome were performed by Fisher’s exact *t* test, and correlation between trichrome and H3K27me3 levels was performed by a PCA and χ^2^ test.

### Study approval.

All animal experiments were performed in accordance with protocols approved by IACUC at the University of Iowa. MPNSTs were obtained from the University of Iowa Department of Pathology with previous approval from the Institutional Review Board (IRB 201408808).

## Author contributions

RDD, QRB, AS, and CSS designed the research studies. QRB, AS, GRM, APV, and CSS conducted experiments. QRB, AS, GRM, APV, ALI, CSS, EAL, GR, VKA, MRT, and WRG acquired data. QRB, AS, CSS, APV, and RDD analyzed data. MRT and BD provided reagents. RDD, QRB, AS, and CSS wrote the manuscript. All authors approved the manuscript.

## Supplementary Material

Supplemental data

Supplemental video 1

Supplemental video 2

## Version 1 09/06/2022

In-Press Preview

## Version 2 10/24/2022

Electronic publication
