# Supplementary material for: PRC2 loss drives MPNST metastasis and matrix remodeling
Source: JCI Insight. 2022 Oct 24;7(20):e157502. doi: 10.1172/jci.insight.157502 (PMC9714789; doi:10.1172/jci.insight.157502)
Supplement: Supplemental data [file jciinsight-7-157502-s042.pdf]

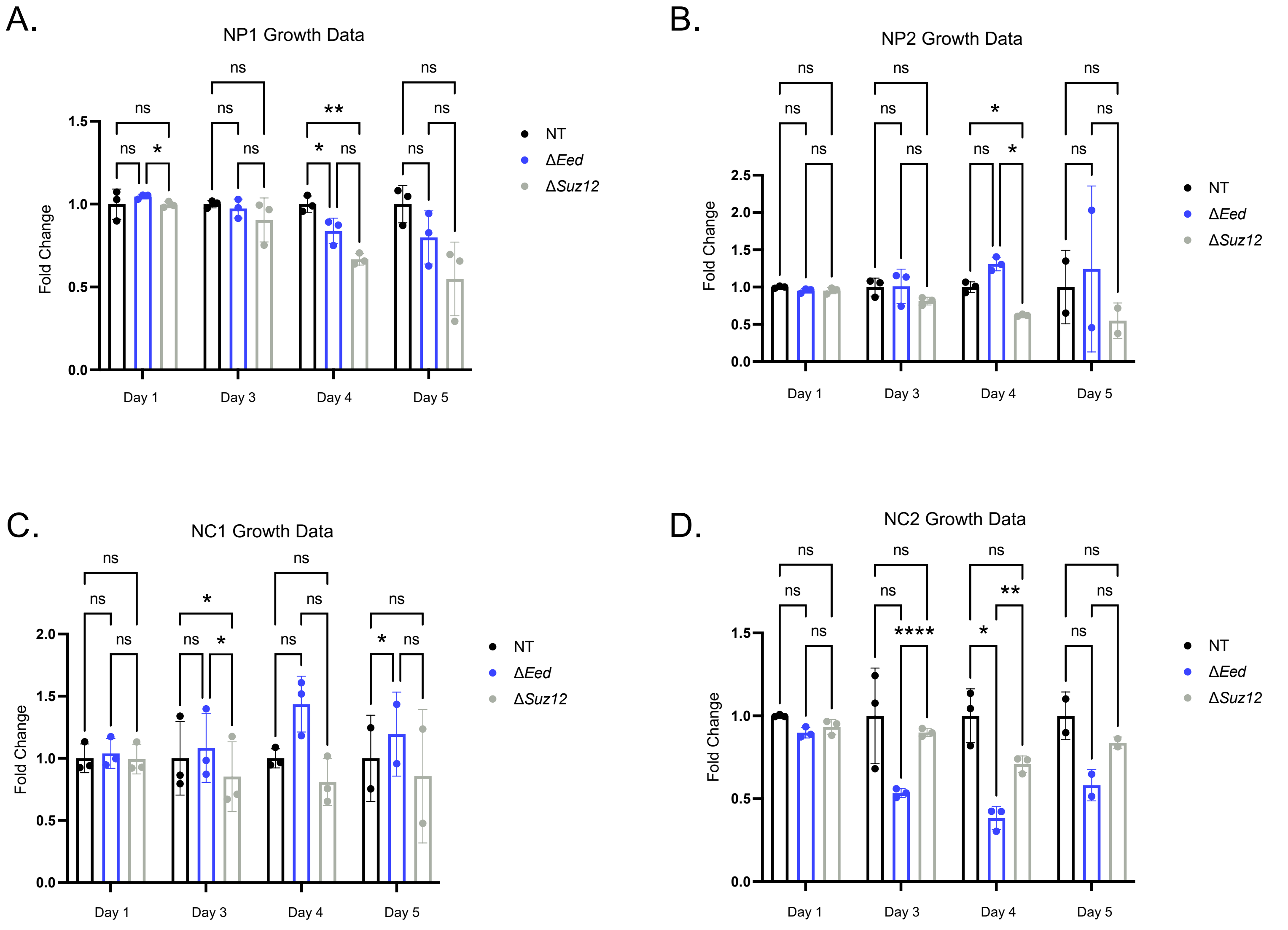

Supplemental Figure 1: ***In vitro* growth.** Longitudinal alamar blue growth assays at 1 day (n=3), 3 days (n=3), 4 days (n=3), and 5 days (n=2) post seeding show minimal differences in cell growth with loss of *Eed* or *Suz12* compared to NT controls in all isogenic series including (A) NP1, (B) NP2, (C) NC1, and (D) NC2. Analyzed by one-way ANOVA with Tukey's multiple comparison. Data represents biological replicates with the mean ± SD; *P < 0.05, **P < 0.01, ***P < 0.001, ****P < 0.0001.

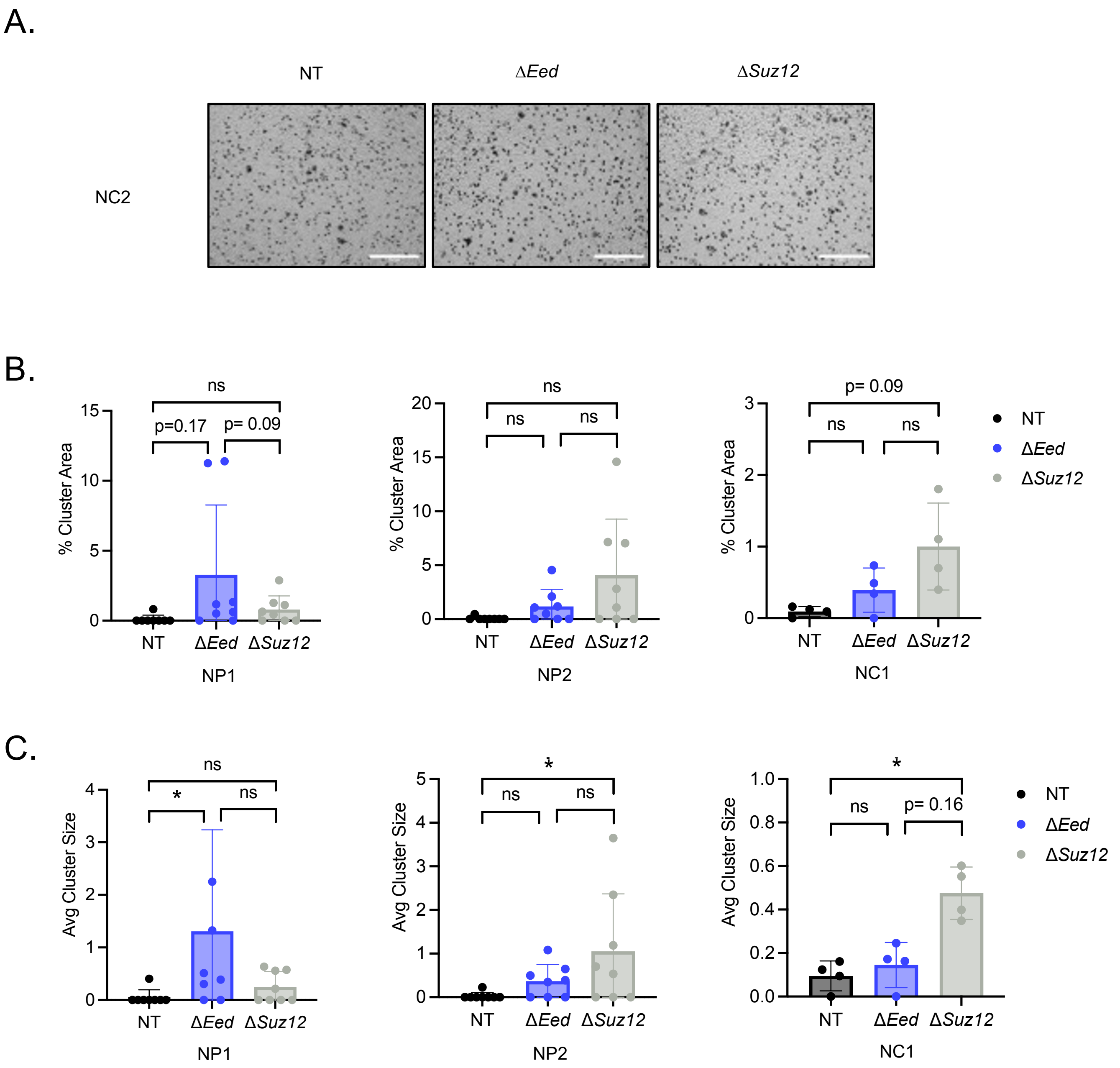

Supplemental Figure 2: Additional cell cluster quantification. (A) Representative images (20x) of NC2 cell panel showing no clustering phenotype observed after transwell invasion (n=4). (B) Quantification of NP1, NP2, and NC1 cell panels cell cluster percent area from transwell invasion assays showing trending increases in cluster area with PRC2 loss (n=4). (C) Quantification of NP1, NP2, and NC1 cell panels average cluster size (cluster area/# of clusters) from transwell invasion assays (n=4). Cluster area were analyzed by Kruskal-Wallis with multiple comparisons. Data represents individual clusters with the mean ± SD; *P < 0.05, **P < 0.01, ***P < 0.001, ****P < 0.0001.

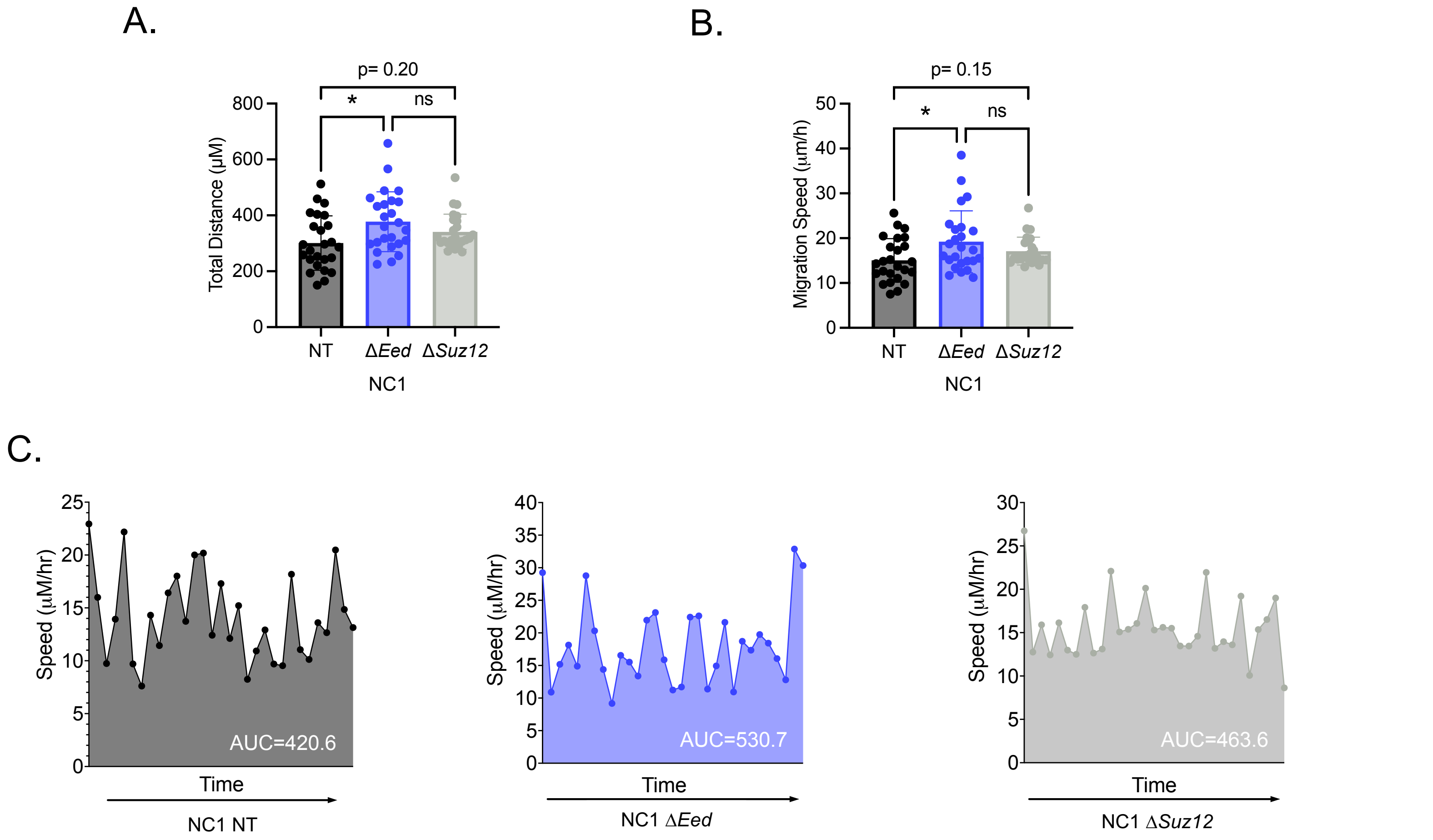

Supplemental Figure 3: Additional cell motility features from time-lapse movie. NC1 cell motility was quantified using ImageJ MTrackJ software. Cell motility tracks (tracks=25) were graphed as total distance travelled (A) and migration speed (B) (n=25). Cells with *Eed* loss showed increased distance travelled and migration speed compared to non-targeting controls. Data was analyzed by One-way ANOVA w/ Tukey's multiple comparison. (C) Quantification of speed over time is calculated with speed (μM/hr) using area under the curve analysis (AUC). Data represents individual clusters from one biological replicate with the mean ± SD; *P < 0.05, **P < 0.01, ***P < 0.001, ****P < 0.0001.

Supplemental Movie: NC1 3D collagen migration phase-contrast movies. Phase-contrast movies of NC1s plated on collagen (35mm^3 dish) for 18hr before capturing cell growth via 5 min increment frames for 20 hours (1200min; 10x) (n=3).

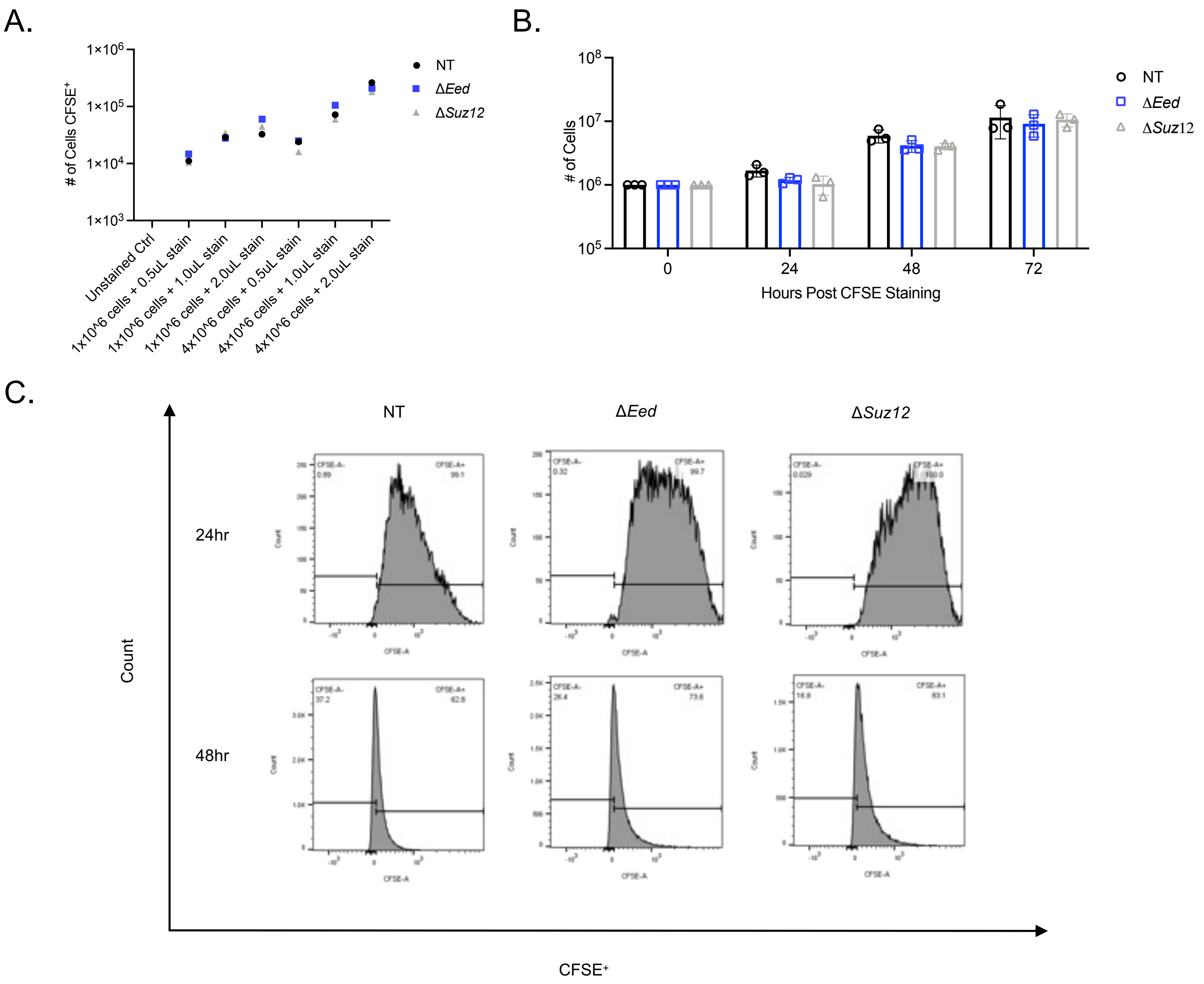

Supplemental Figure 4: CFSE staining of MPNST cells is similar across all PRC2 genotypes. (A) Optimization of CFSE+ cells at various cell and label concentrations. (B) Cell viability at 0hr, 24hr, 48hr, and 72hr post CFSE staining ($1.0x10^6$ cells/mL, 1.0uL of CFSE stain/mL of cells [5µM] via trypan exclusion (n=3). (C) Number of CFSE$^+$ cells following CFSE staining with previous concentrations at 24hr and 48hr post staining. Data was analyzed by One-way ANOVA w/ Tukey's multiple comparison. Data represents biological replicates with the mean ± SD; *P < 0.05, **P < 0.01, ***P < 0.001, ****P < 0.0001.

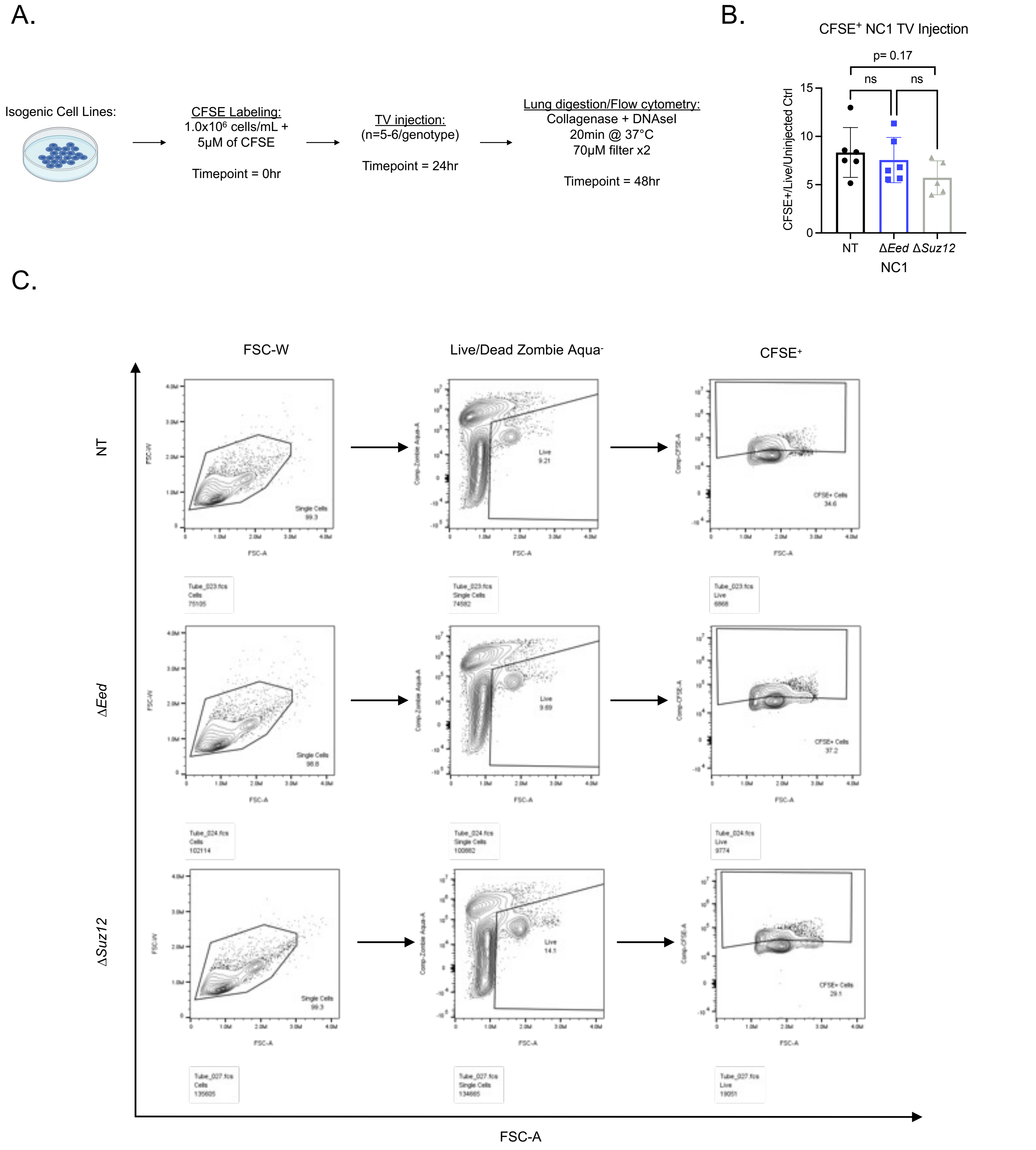

Supplemental Figure 5: PRC2 status does not alter initial number of CFSE-labeled MPNST cells in the lung following tail vein injection. (A) Schematic of cell staining, tail vein injection, and harvesting. (B) Number of CFSE+ MPSNT cells present in the the lung of NSG mice 24 hours after tail vein injection (n=5-6 mice per genotype). (C) Gating scheme for flow cytometric analysis of CFSE⁺ NC1 isogenic cell line panel (NT, n=6; Δ*Eed*, n=6; Δ*Suz12*, n=5). Data was analyzed by One-way ANOVA w/ Tukey's multiple comparison. Data represents biological replicates with the mean ± SD; *P < 0.05, **P < 0.01, ***P < 0.001, ****P < 0.0001.

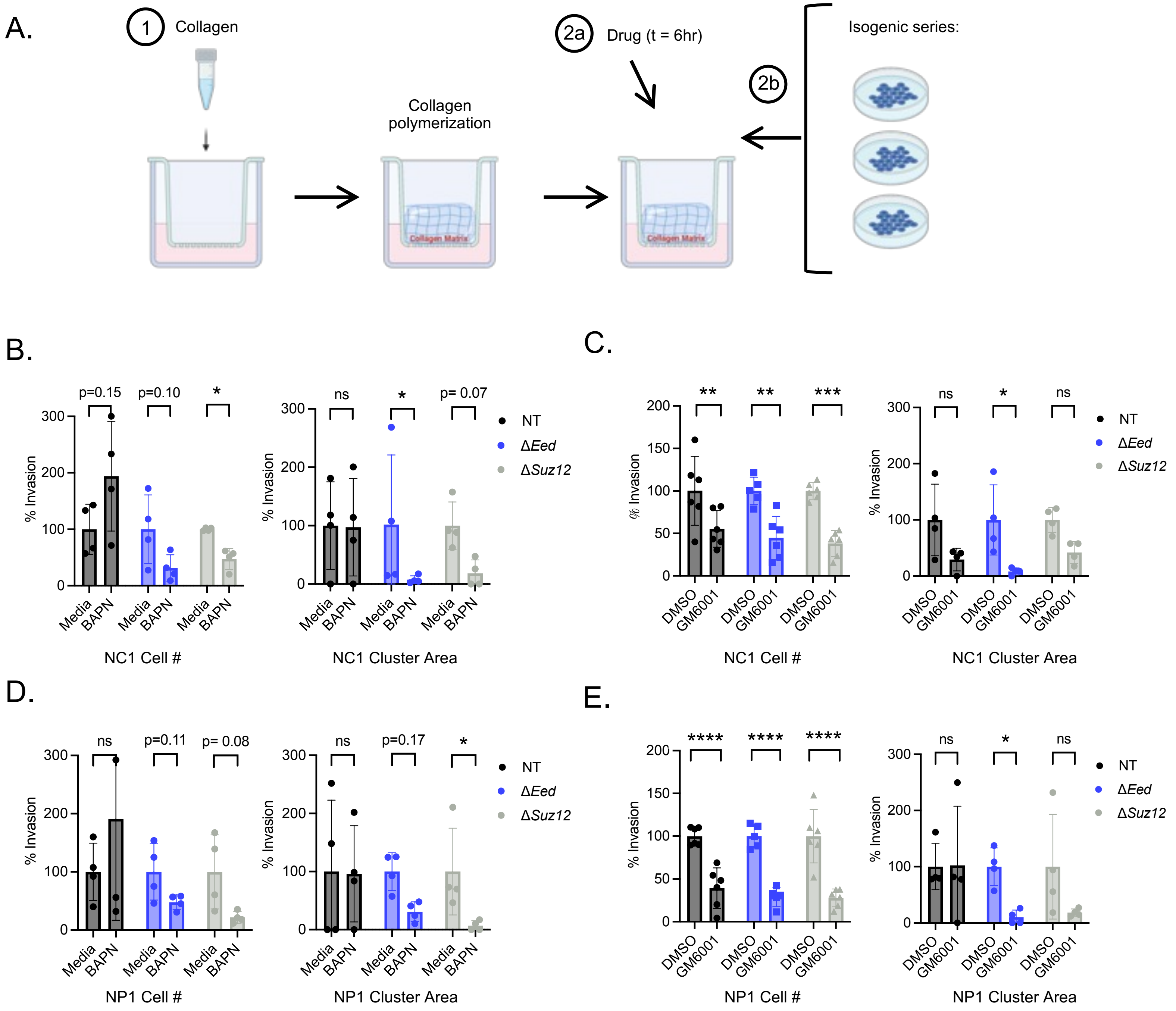

Supplemental Figure 6: Pharmacological inhibition of LOX enzyme function, but not MMP function, decreases PRC2-related metastatic-associated phenotypes. (A) Schematic of transwell invasion across collagen with LOX and MMP inhibitor treatment. (B, D) Treatment of NC1 (B) or NP1 (D) cells with the pan Lysl-oxidase inhibitor BAPN (400µM) for 6 hours decreases cell invasion and cluster formation only in cells with Eed or Suz12 loss (n=4). (C, E) Treatment of NC1 (C) or NP1 (E) cells with pan-MMP inhibitor GM6001 (50µM) for 6 hours decreases cell invasion and cluster formation in both PRC2 wildtype and PRC2-deleted cells (n=4-6). The effects of inhibitor treatment were quantified as relative invasion (cell number treatment vs control; left) and relative cell clustering (total area; right). Data analyzed by student's t-test (cell number) or beta-regression (cluster area). Data represents biological replicates with the mean ± SD; *P < 0.05, **P < 0.01, ***P < 0.001, ****P < 0.0001.

A.

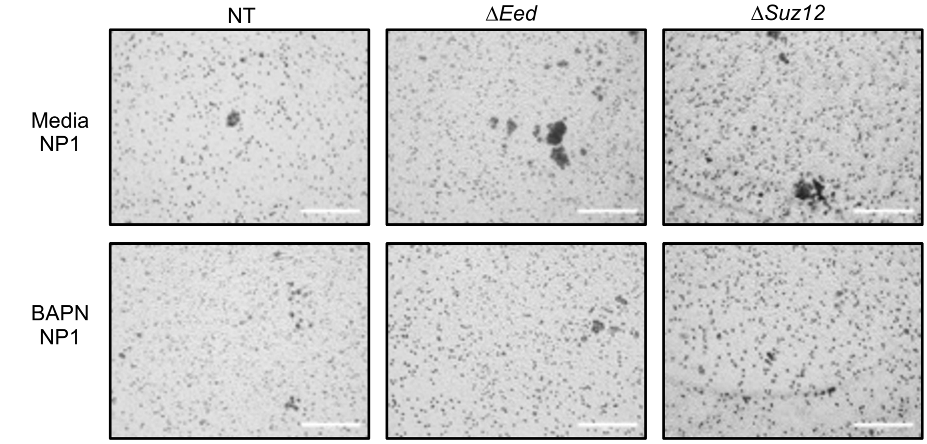

B.

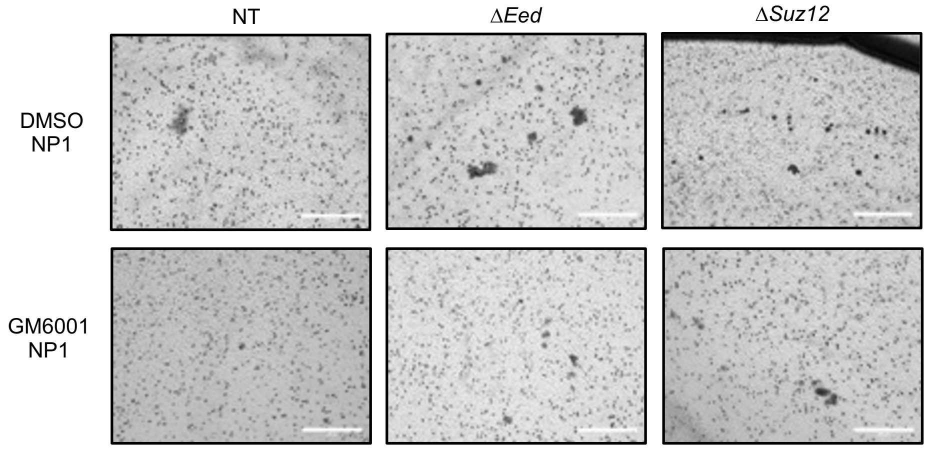

Supplemental Figure 7: Images of PRC2-deleted cells following treatment with LOX and MMP inhibitors. Representative images (20x) of cell clustering of the NP1 (n=4) isogenic cell lines after 6-hour treatment with (A) the LOX inhibitor BAPN or media control or (B) the MMP inhibitor GM6001 or DMSO control. Data represents biological replicates with the mean ± SD; *P < 0.05, **P < 0.01, ***P < 0.001, ****P < 0.0001.

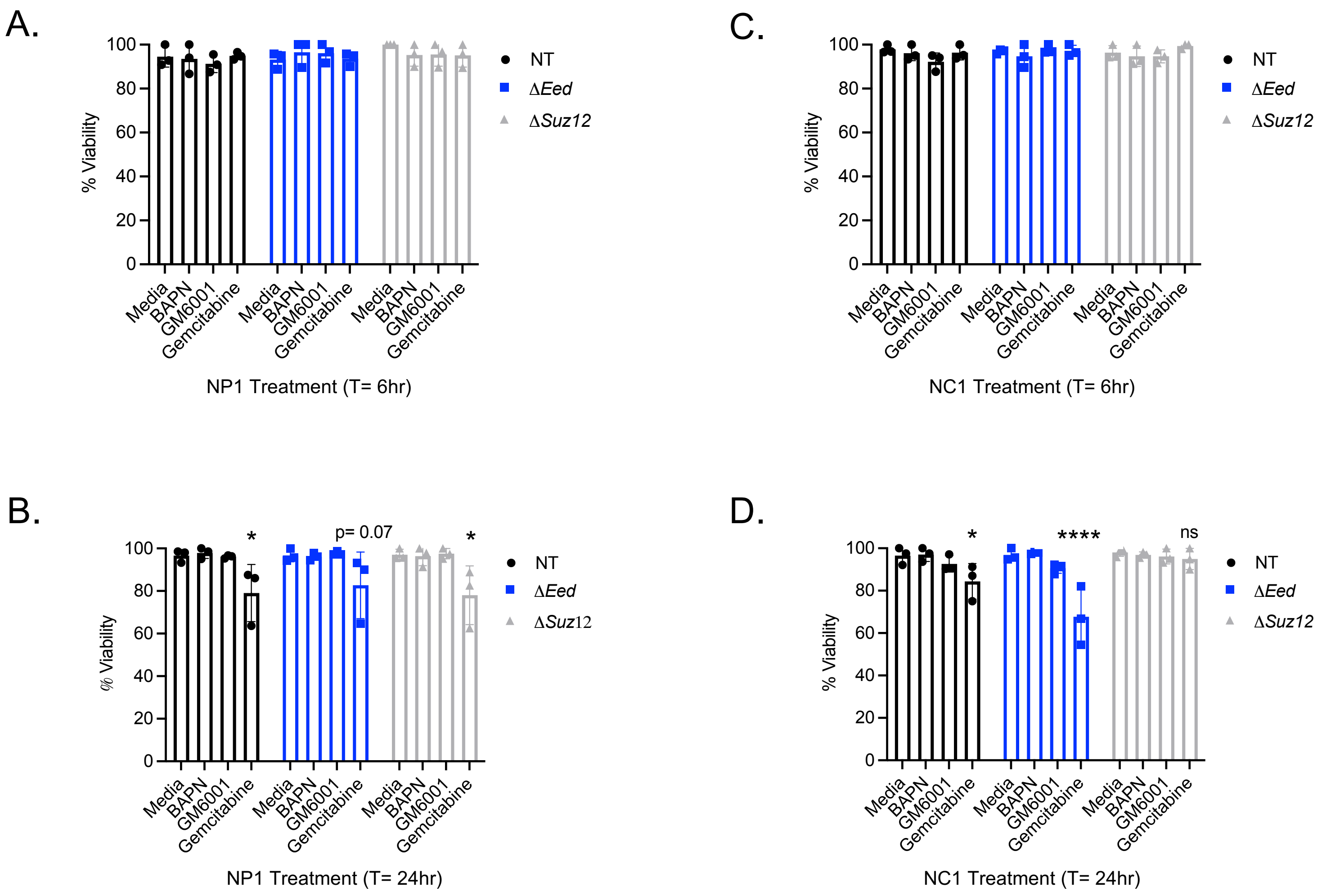

Supplemental Figure 8: Viability of MPNST cells following GM6001 and BAPN treatment. (A-B) NP1 cell viability at 6 hr (A) and 24 hr (B) after treatment with media alone, BAPN, GM6001, or gemcitabine as a positive control. (C-D) NC1 cell viability at 6 hr (A) and 24 hr (B) after treatment with media alone, BAPN, GM6001, or gemcitabine as a positive control. Quantified via trypan exclusion (n=3). Data was analyzed by One-way ANOVA w/ Tukey's multiple comparison. Data represents biological replicates with the mean ± SD; *P < 0.05, **P < 0.01, ***P < 0.001, ****P < 0.0001.

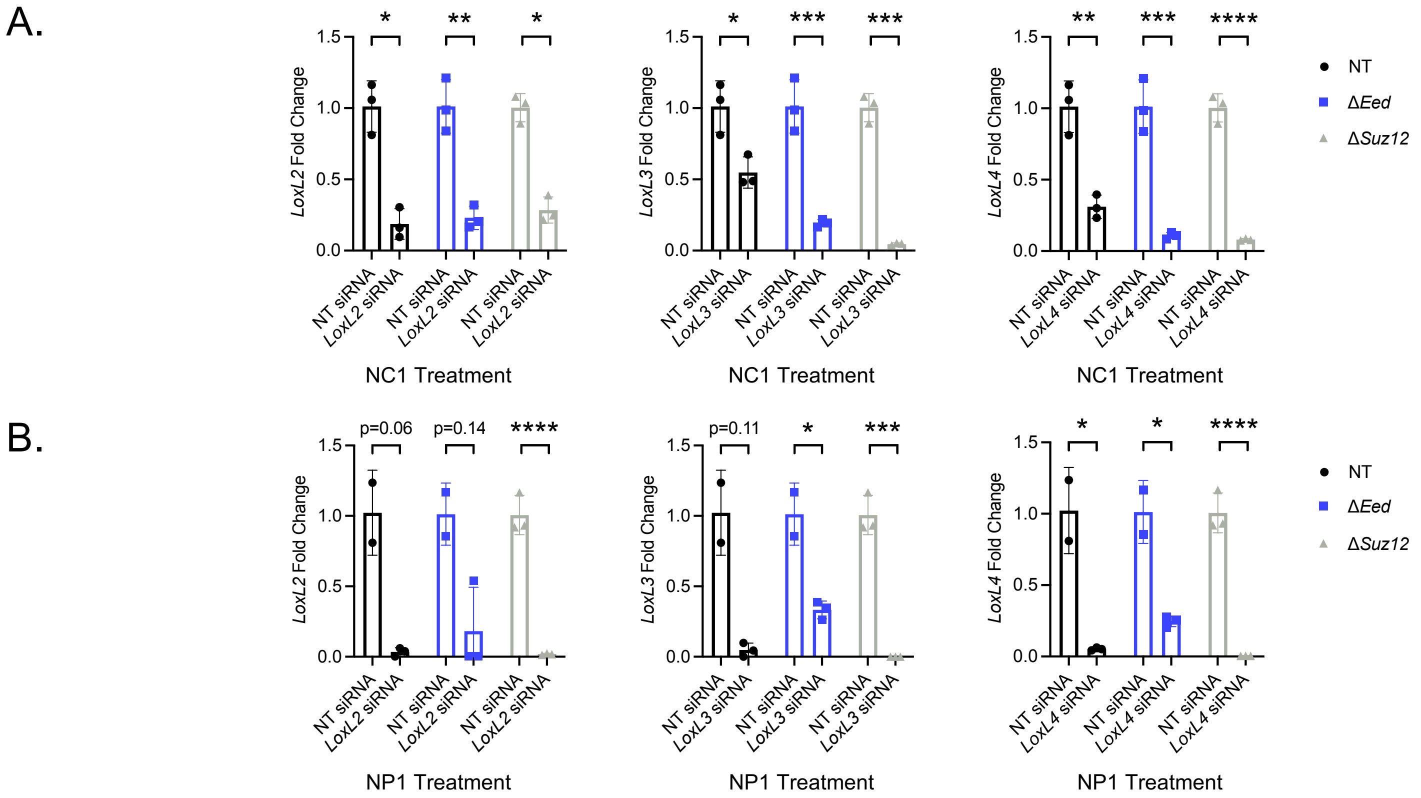

Supplemental Figure 9: Lox family siRNA qRT-PCR. Representative *LoxL2*, *LoxL3*, and *LoxL4* gene expression by quantitative RT-PCR in (A) NC1 and (B) NP1 isogenic cell lines shows downregulation of targets when transfected with respective *Lox* family siRNA (50nm) compared to non-targeting (NT) control siRNA. qRT-PCR data analyzed by Welch's T-test. Data is representative of biological replicates (n=3) with the mean ± SD; *P < 0.05, **P < 0.01, ***P < 0.001, ****P < 0.0001.

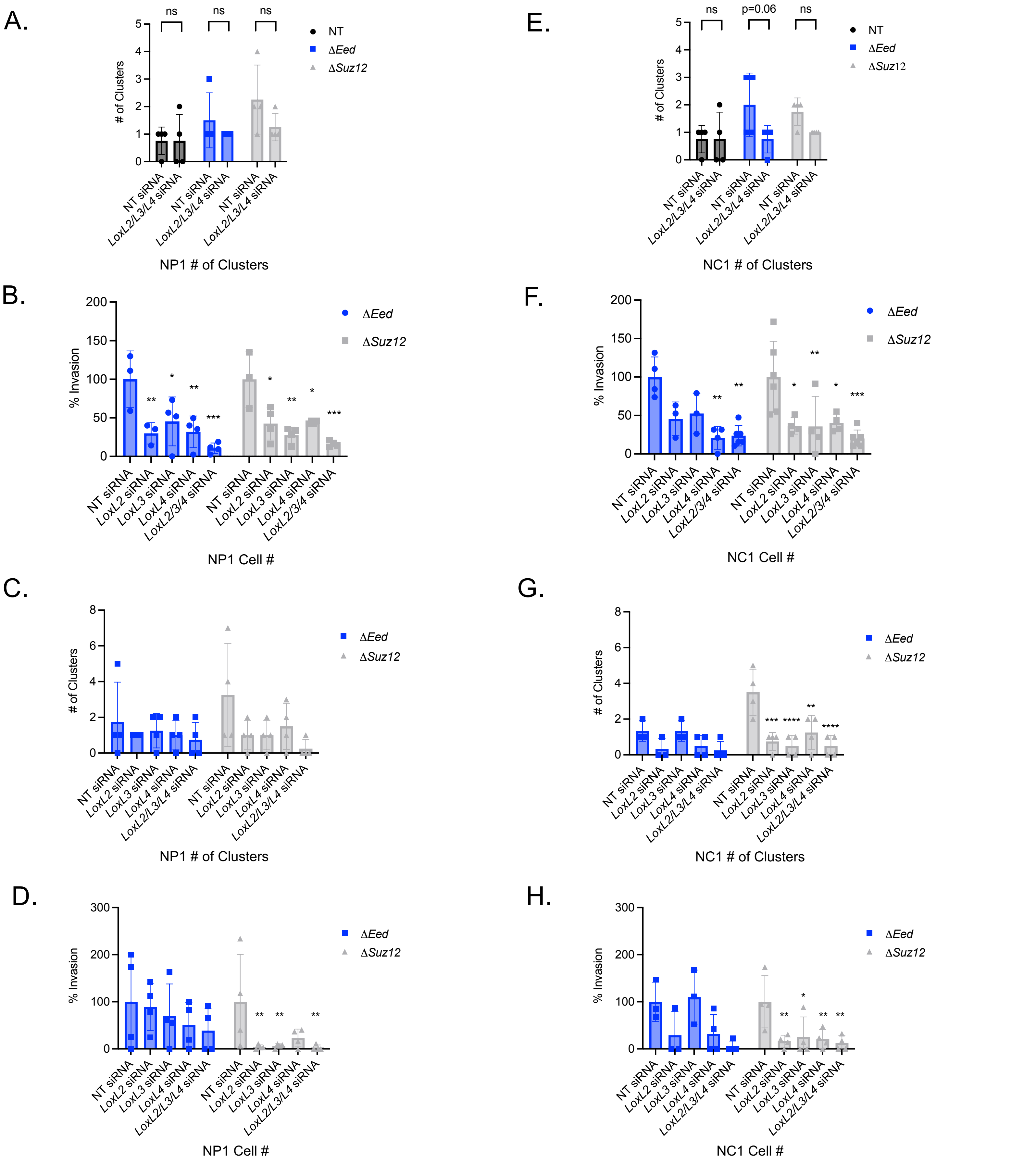

Supplemental Figure 10: single siRNA transwell and additional cluster normalization. (A) Transwell invasion # of clusters of NP1 cells following knockdown of pooled *Lox* family members compared to NT siRNA (n=3-4). (B-D) Transwell invasion cell # (B), # of clusters (C), and cluster area (D) of NP1 cells following knockdown of individual *Lox* family members compared to NT siRNA (n=3-4). (E) Transwell invasion # of clusters of NC1 cells following knockdown of pooled *Lox* family members compared to NT siRNA (n=3-4). (F-H) Transwell invasion cell # (F), # of clusters (G), and cluster area (H) of NC1 cells following knockdown of individual *Lox* family members compared to NT siRNA (n=3-4). Transwell data analyzed by One-way ANOVA w/ Tukey's multiple comparison. Data represents biological replicates (A-B, E-F) and individual clusters (C-D, G-H) with the mean ± SD; $*P < 0.05$, $**P < 0.01$, $***P < 0.001$, $****P < 0.0001$.
